# Egg microbiome of the yellow-spotted Amazon river turtle (*Podocnemis unifilis*) modulates fusariosis fungal infection and hatching success

**DOI:** 10.1038/s42003-026-10404-8

**Published:** 2026-06-04

**Authors:** Ana Sofia Carranco, David Romo, Maria de Lourdes Torres, Kerstin Wilhelm, Mark A. F. Gillingham, Simone Sommer

**Affiliations:** 1 Institute of Evolutionary Ecology and Conservation Genomics, University of Ulm, Ulm, Germany; 2 Tiputini Biodiversity Station, Universidad San Francisco de Quito, Diego de Robles y Via Interoceanica s/n, Quito, Ecuador; 3 Laboratorio de Biotecnología Vegetal, Universidad San Francisco de Quito USFQ, Diego de Robles y Via Interoceanica s/n, Quito, Ecuador; 4 Department of Ornithology, Max Planck Institute for Biological Intelligence, Seewiesen, Germany

**Keywords:** Microbial ecology, Microbial ecology

## Abstract

Turtle egg fusariosis, caused by pathogens of the *Fusarium solani* species complex (FSSC), threatens turtle populations globally, through embryonic mortality and hatching failure. Host–associated microbiomes, including the bacteriome and mycobiome, are hypothesized to mediate disease outcomes in oviparous vertebrates, but this remains largely unexplored. Here, we characterised the inner–egg bacteriome and mycobiome of uninfected and FSSC–infected eggs at three developmental stages in the vulnerable yellow–spotted Amazon river turtle (*Podocnemis unifilis*). Lower mycobiome evenness was strongly associated with increased FSSC infection propensity and intensity. Regardless of infection status, higher microbial diversity positively correlated with hatching success, and hatched eggs showed more complex interkingdom interactions. We also identified bacterial and fungal genera whose relative abundance was negatively associated with FSSC infection. These findings support the hypothesis that the egg microbiome may influence infection and hatching outcomes, with implications for microbiome–informed conservation strategies for threatened turtle populations.

## Abstract (web summary)

Inner eggshell microbiota in the yellow-spotted Amazon river turtle support improved hatching success and reduced *Fusarium* infection, highlighting the potential for microbiome-based conservation.

## Introduction

The emergence of fungal diseases, including white–nose syndrome in bats, chytridiomycosis in amphibians and egg fusariosis in turtles, represents a significant threat to biodiversity, contributing to the ongoing sixth mass extinction^1,2^. In view of their pathogenicity to humans, plants and wildlife, *Fusarium* trans–kingdom pathogens have been classified as high–priority pathogens by the World Health Organization in 2022^3^. Turtle egg fusariosis, caused by pathogens belonging to the *Fusarium solani* species complex (FSSC), represents a significant threat to marine turtle populations around the globe^4–7^. Furthermore, the disease has recently been observed in a freshwater turtle species inhabiting a pristine region of the Amazon basin^8^. FSSC pathogens infect eggs during the incubation period, resulting in high rates of embryo mortality and hatching failure^4,5^. The capacity of fungal pathogens to persist in the environment without a host and to infest a vast array of species renders their detection and control particularly challenging from a conservation perspective^1^. Moreover, the host–protective traits of turtles eggs that may confer resistance to fungal infections remain to be elucidated.

The host microbiome plays a key role in pathogen resistance^9–12^. Commensal microorganisms can prevent pathogens from colonising the host through competitive exclusion^13^. Furthermore, specific commensal bacteria can produce antifungal metabolites that inhibit fungal infections^14^. While the majority of microbiome research has focused on the bacterial community, also known as the bacteriome, the fungal community, termed the mycobiome, has been largely overlooked. Nevertheless, recent studies suggest that the mycobiome may also play a crucial role in maintaining host health^15^. In the context of fungal diseases in wildlife, the mycobiome has been shown to confer tolerance to white–nose syndrome in bats^16^. However, the precise mechanisms by which the host–associated microbiome contributes to the suppression of fungal diseases remain unclear. In particular, the role of the egg–associated microbiome in mediating pathogen resistance and its relationship with hatching success are areas that have received little attention, including in oviparous reptiles. A recent study on the lizard *Sceloporus virgatus* demonstrated that bacteria, vertically transmitted via the maternal cloaca, colonise the eggshell, enhance resistance to fungal infections and promote hatching success^17^. Vertical transmission has also been observed in turtles^18^, but the role of the eggshell microbiome in pathogen resistance remains unknown in this taxon. We therefore hypothesise that the inner eggshell microbiome of turtles may play a pivotal role in determining *Fusarium* resistance and hatching success^19,20^.

In this study, we used the yellow–spotted Amazon river turtle (*Podocnemis unifilis*) as a model to investigate the potential role of the bacteriome and mycobiome of the inner eggshell in fungal pathogen defence and hatching success. We have previously demonstrated that eggs of the yellow–spotted Amazon river turtle are susceptible to egg fusariosis^8^ and possess an inner bacteriome that is primarily derived but distinct from the nest environment^20^. The inner–egg bacteriome is, in turn, the dominant source of hatchling cloacal bacteriome^20^. Specifically, for this study, we sought to determine whether inner–egg bacteriome and mycobiome diversity predict FSSC prevalence and infection intensity. Diverse microbiomes may provide resistance against fungal pathogens through competitive exclusion and complex interkingdom interactions^13,14^. Conversely, pathogens may outcompete commensals, reducing microbiome diversity. Furthermore, we investigated whether the diversity and composition of the bacteriome and mycobiome differ between eggs that have undergone arrested development at an early and late embryonic stage compared to eggs that have successfully hatched. We subsequently investigated whether differences in inner egg bacteriome and mycobiome are predictors of hatching success. We aimed to identify the specific microbial genera, predicted functional profiles and interkingdom network interactions that were linked with FSSC susceptibility and hatching success. Our findings suggest that both the egg bacteriome and mycobiome are critical in modulating resilience to turtle egg fusariosis and hatching success.

## Results

### Sequencing results

For the 16S rRNA gene, pre–processing with QIIME2 recovered a total of 7,292,052 reads with an average of 56,969 per sample. After excluding samples with fewer than 10,000 reads, we retained 121 samples, each with a minimum sequencing depth of 13,912 reads. For the ITS1 and ITS2 regions, we recovered a total of 2,025,198 reads across 114 samples, with an average of 18,411 per sample. After excluding samples with fewer than 3000 reads, we retained 109 samples with a minimum sequencing depth of 3300 reads. Finally, for the TEF alpha gene, we recovered 1,250,334 reads in 104 samples, with an average of 9693 reads per sample. From the TEF alpha data, we extracted FSSC–positive samples based on ASV counts of *F. solani* and *F. keratoplasticum* greater than one and incorporated FSSC prevalence and abundance as variables in the bacteriome and mycobiome metadata.

### Mycobiome diversity negatively predicts fusariosis

**Fig. 1:**
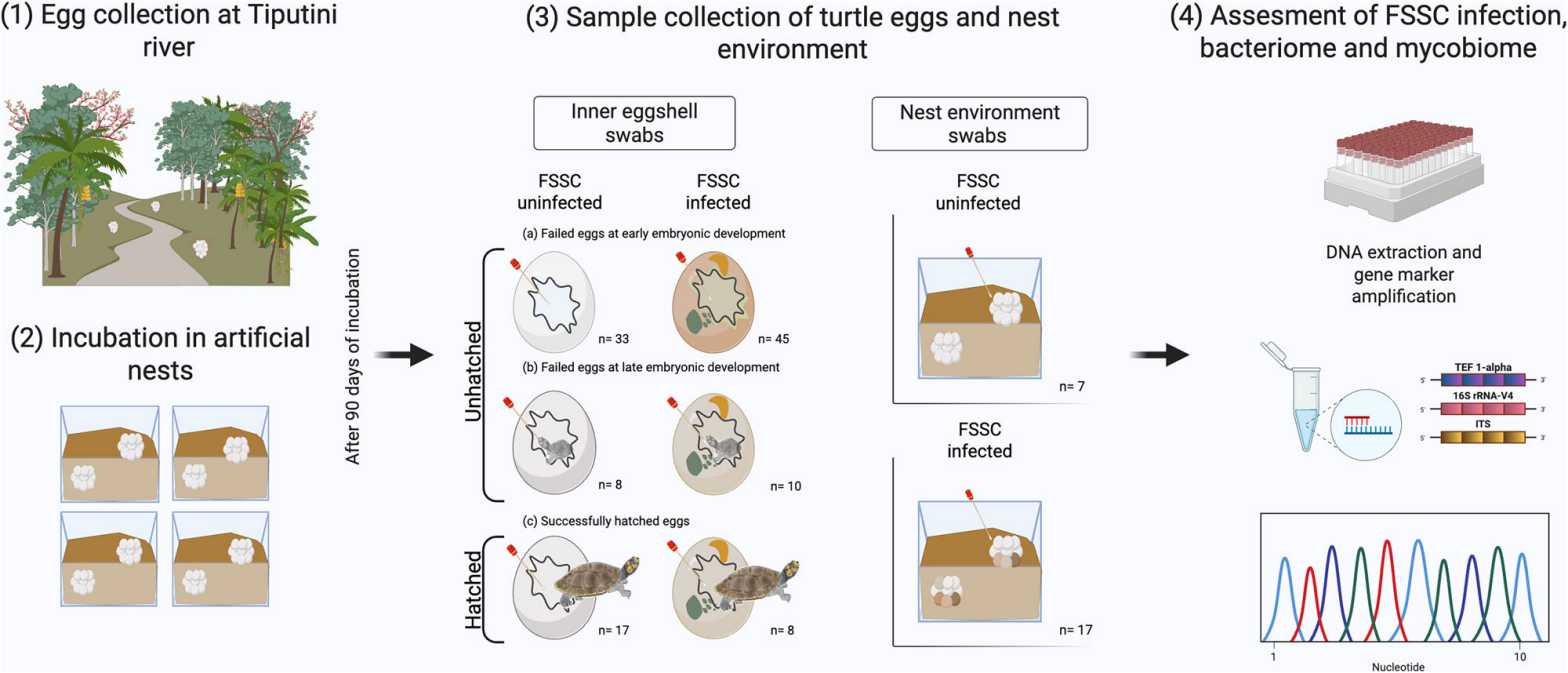
Outline of the study design of the fieldwork conducted for the present project. 1. Eggs were collected at various nesting sites along the Tiputini River, within the Yasuni Biosphere Reserve in Ecuador. 2. Eggs were subsequently transported to artificial nests at the Tiputini Biodiversity Station (TBS). 3. Samples were taken from the inner egg shells of unhatched and hatched eggs classified into three categories: **a** dead at an early developmental stage (less than 30 days of incubation, labelled as failed early development (E.D.)), **b** failed to hatch within 100–120 days of incubation with an embryo developed to a late developmental stage (failed late development (L.D.)), and **c** successfully hatched (hatched eggs); as well as soil samples from 24 successfully sequenced (from 31 sampled) artificial nests. Sample sizes are given (*n*); 4. Transport of samples from Ecuador to Germany for *Fusarium*, bacteriome, and mycobiome metabarcoding and high-throughput sequencing. The figure was created in BioRender. Sommer, S. (2026) https://BioRender.com/oe14ebm. Turtle drawing in the figure created by Ana S. Carranco (author).

After a 90–day incubation period in 31 artificial nests, a total of 121 eggs of *P. unifilis* were screened and the developmental status of the eggs was determined and categorised into three stages: eggs that died at an early developmental stage (less than 30 days of incubation, henceforth termed failed E.D.), eggs that failed to hatch after 100–120 days of incubation with a viable developed embryo at a late developmental stage (henceforth termed failed L.D.), and successfully hatched eggs (Supplementary Table 1). Sixty–three eggs from 24 nests were found to be infected with two members of FSSC, specifically *F. solani* and *F. keratoplasticum* (Supplementary Table 1, Fig. 1). From the 121 screened eggs, we obtained bacteriome and mycobiome sequences for 121 and 109 eggs, respectively (Fig. 1, Supplementary Table).

**Fig. 2:**
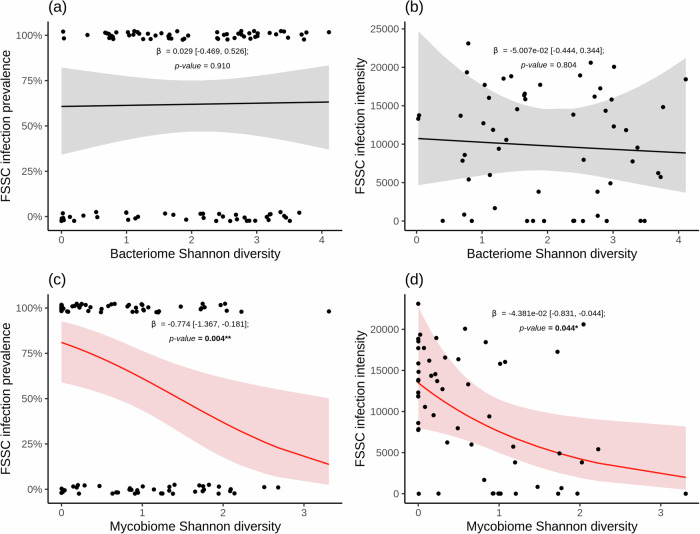
Association of bacterial and fungal diversity in turtle eggs with fusariosis infection. Shown are FSSC infection prevalence (in %) and FSSC infection intensity (zero–truncated ASV abundance of FSSC based on target Illumina sequencing of the TEF alpha gene using *Fusarium*–specific primers) according to (**a**, **b**) bacteriome and (**c**, **d**) mycobiome Shannon diversity. The fitted line and 95% confidence intervals (shaded area) were estimated using a GLMM with nest ID as a random factor. Jitter is applied to the data points to show the repeated observations. Significant relationships are represented in red.

Using generalised linear mixed models (GLMM) with NestID as a random factor to account for the non–independence of sampling multiple eggs from the same nest (i.e. to avoid pseudo–replication), we investigated the potential association of *Fusarium* prevalence and intensity with bacterial and fungal diversity. The response variables were FSSC infection status (binomial matrix, 0 = uninfected, 1 = infected) and FSSC ASV abundance (zero–truncated ASV counts of *F. solani* and *F. keratoplasticum* strains based on targeted Illumina sequencing of the TEF alpha gene using *Fusarium*–specific primers). To avoid collinearity, alpha and beta diversity metrics (species richness, Shannon index and beta diversity dispersion) were entered as explanatory variables in separate independent models. Bacteriome Shannon diversity was not associated with the probability of FSSC infection (*β* = 0.029, 95% CI [−0.469, 0.526], df = 1, *χ*2 = 0.013, *p*–value = 0.910; Fig. 2a) nor intensity (*β* = −0.050, 95%CI [−0.444, 0.344], df = 1, *χ*2 = 0.062, *p*–value = 0.804; Fig. 2b). Similarly, no significant effect was observed for mycobiome species richness or community dispersion (beta diversity) on FSSC infection probability (Supplementary Fig. 1, Supplementary Table 2) or intensity (Supplementary Table 3). However, a strong negative effect of mycobiome Shannon diversity on FSSC infection probability (*β* = −0.774; 95% CI [−1.367, −0.181], df = 1; *χ*2 = 8.385; *p*–value = 0.004; Fig. 2c) and intensity (*β* = -0.438; 95% CI [−0.832, −0.045], df = 1; *χ*^2^ = 4.058; *p–value* = 0.044; Fig. 2d) was observed. The random factor Nest ID was also significant, indicating that some clutches were consistently more susceptible to *Fusarium* infection than others (Nest ID; *χ*2 = 4.959; p–value = 0.026). As has been previously documented in this population, the probability of infection varies considerably between nests^8^. Overall, our results suggest that either increased mycobiome diversity in the inner–egg confers resistance to FSSC infection or that the infection reduces mycobiome diversity.

### Healthy embryonic development is linked with microbial diversity and composition

**Fig. 3:**
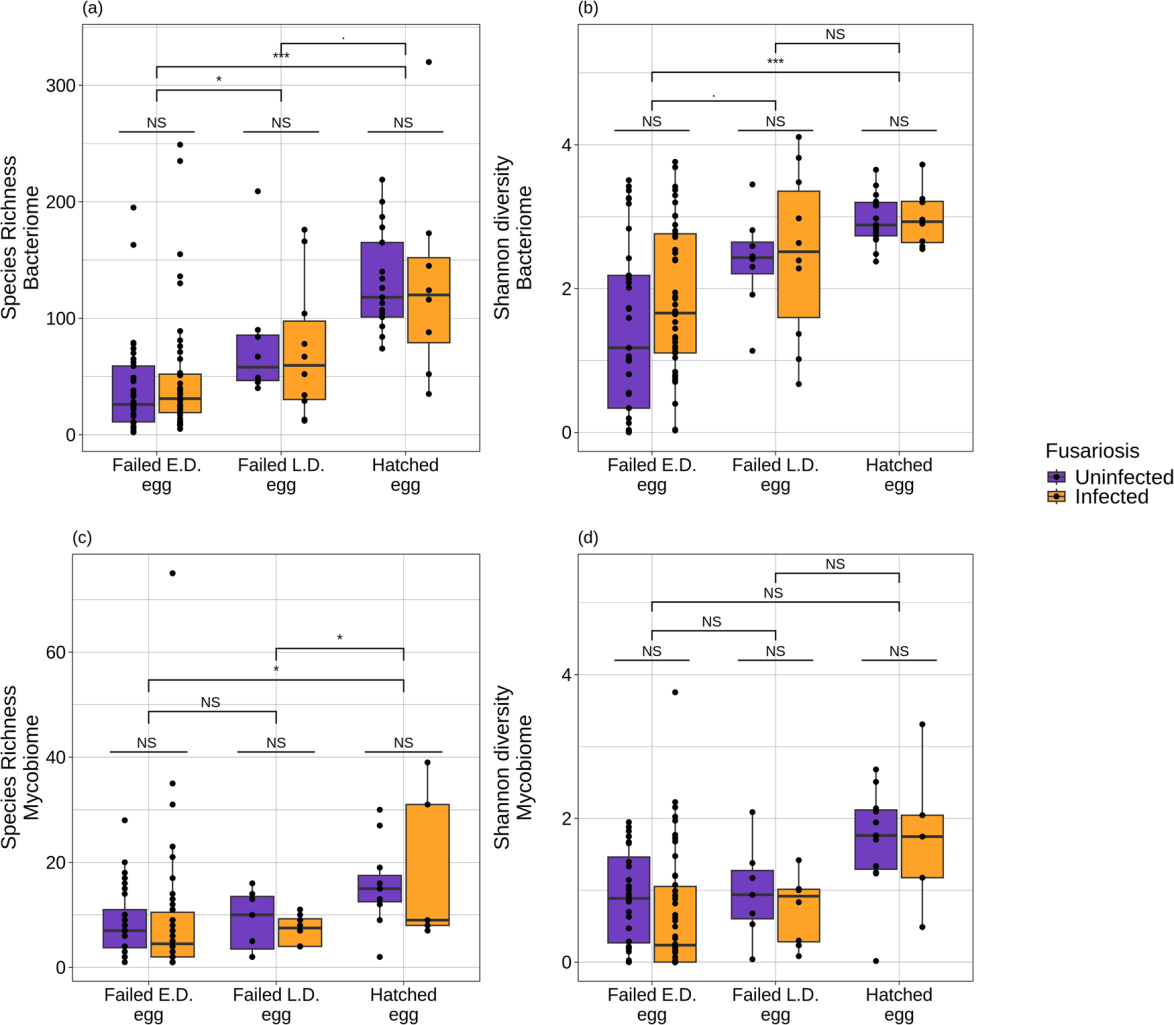
Bacterial and fungal diversity at three distinct embryonic developmental stages according to *Fusarium* infection. The boxplots illustrate the alpha diversity indices (species richness and Shannon diversity) of the internal egg bacteriome and mycobiome. **a** Bacterial species richness, **b** bacterial Shannon diversity, **c** fungal species richness and **d** fungal Shannon diversity according to the developmental status of the eggs: failed early embryonic development (failed E.D. eggs), failed late embryonic development (failed L.D. eggs) and successfully hatched eggs (hatched eggs), as well as the fusariosis infection status (FSSC uninfected or infected). A post–hoc test with Tukey’s multiple comparison correction of the general linear models was employed to ascertain whether there were differences among the three developmental stages and fusariosis infection status. Groups with significant differences are shown with an (*) according to the adjusted *p*–value (“***” <0.001; “**” <0.01; “*” <0.05; “.” <0.1) and groups with no significant differences (>0.1) are indicated by the acronym N.S. (not significant).

We next investigated whether variations in bacterial and fungal diversity within the inner egg were associated with different stages of embryonic development and tested for differences in the interaction between egg development and fusariosis infection status. Using general linear models (GLM), we first tested for differences in alpha diversity of the bacteriome and mycobiome of internal eggs according to egg developmental stage and fusariosis infection status. We observed significant differences in species richness of both bacteria and fungi, and bacterial Shannon diversity according to developmental status (bacteriome species richness: LRT_2, 117_ = 33.394, *p–value* < 0.001, Fig. 3a; mycobiome species richness: LRT_2, 105_ = 9.163, *p–value* = 0.010, Fig. 3c; bacteriome Shannon index: F_-2, 119_ = 12.603, *p–value* < 0.001, Fig. 3b) but mycobiome Shannon diversity was not significantly different (*χ*^2^ = 4.886; *p–value* = 0.087; Fig. 3d). Post–hoc analyses using Tukey’s test revealed that bacterial and fungal species richness, as well as bacteriome Shannon diversity, were significantly reduced in failed E.D. eggs (*p–value* < 0.001; Fig. 3a, b, and c), and that bacterial and fungal diversity of failed L.D. eggs was intermediate between that of failed E.D. and hatched eggs (Fig. 3a–c). FSSC infection status, as well as the interaction term between microbial diversity and FSSC infection status, were not significant predictors of any of the alpha diversity metrics (Fig. 3, Supplementary Table 4, Supplementary Table 5, p > 0.05). Overall, our results show that eggs that successfully hatched exhibited a greater diversity of bacterial and fungal species compared to those that failed to develop, particularly at an early developmental stage, suggesting that the inner egg harbours an intricate microbiome vital for healthy embryonic development.

**Fig. 4:**
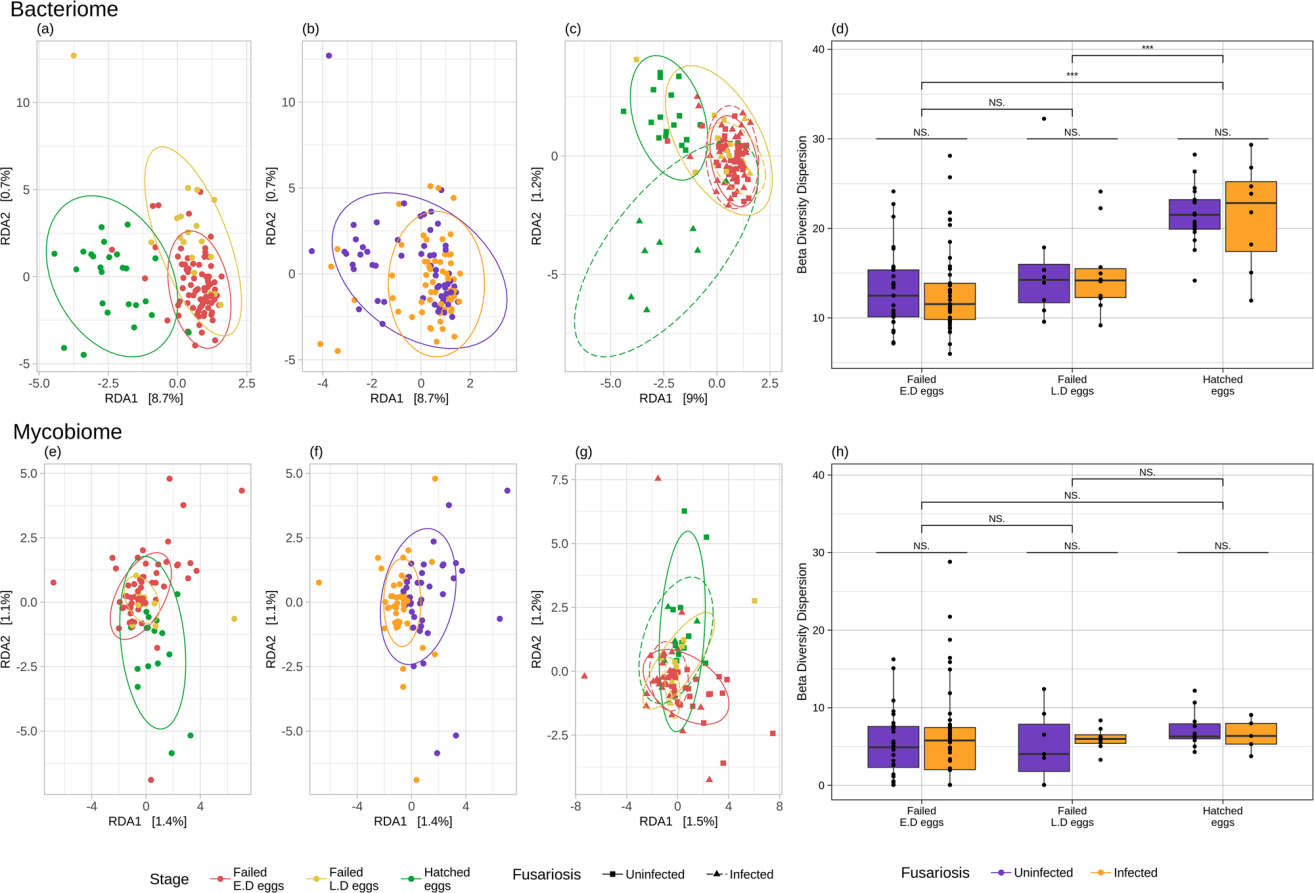
Bacterial and fungal composition of inner eggshells in relation to the egg developmental stage and *Fusarium* infection. Redundant analysis (RDA) ordination plots with Euclidean distances of the beta diversity of bacteriomes (upper panels) and mycobiomes (lower panels) according to (**a**, **e**) egg developmental stage (failed E.D. (red), failed L.D. (yellow), hatched egg (green)), (**b**, **f**) FSSC infection status (uninfected (purple), infected (orange)), (**c**, **g**) the interaction between stage*FSSC, as well as (**d**, **h**) box plots of community dispersion using Turkey’s multi comparison test for the GLS models. Groups with significant differences are shown with an (*) according to the adjusted *p*–value (“***” <0.001; “**” <0.01; “*” <0.05; “.” <0.1) and groups with no significant differences (>0.1) are indicated by the acronym N.S. (not significant).

Subsequently, we investigated whether the bacterial and fungal composition among individuals (beta diversity) differed according to egg developmental status and fusariosis infection, as well as their potential interactions. Redundancy analyses (RDA) were conducted using centred log ratios (rCLR) to account for the compositional nature of the data^21^, and ordination plots were generated using Euclidean distances. Bacterial composition was significantly explained by egg development status (Supplementary Table 6a; F_2,118_ = 6.07; *p–value* < 0.001; RDA adjR2 = 7%, Fig. 4a) and only weakly associated with fusariosis infection prevalence (Supplementary Table 6b; F_1,119_ = 1.35; *p–value* = 0.081; adjR^2^ = 0.2%, Fig. 4b). In addition, when we accounted for the interaction term in the RDA model, significant differences were explained by the interaction between egg development and infection status (Supplementary Table 6c; F_5_,_115_ = 3.17; *p–value* < 0.001; RDA adjR^2^ = 8%, Fig. 4c), where the effect of fusariosis infection on bacterial composition was particularly strong for hatched eggs compared to failed E.D. and L.D. eggs (Supplementary Table 7c and Fig. 4c). Differences in mycobiome composition were not explained by egg development (Supplementary Table 8a; F_2_,_106_ = 0.918; *p–value* = 0.56, Fig. 4e) nor by the interaction term between egg development and infection status (Supplementary Table 8c; F_5_,_103_ = 0.94; *p–value* = 0.56, Fig. 4g). In contrast, variation in mycobiome composition was significantly explained by fusariosis infection status (Supplementary Table 8b; F_1,107_ = 1.55; *p–value* = 0.001; RDA adjR2 = 0.5%, Fig. 4f). These results are consistent with alpha diversity results and indicate that bacteriome composition is a critical factor for healthy egg development and that FSSC pathogens influence both bacteriome and mycobiome composition.

Host–microbiome theory predicts that microbiomes that are perturbed by an infection will be more dissimilar to each other than healthy microbiomes, as each community is expected to respond differently and uniquely to a perturbation, the so–called Anna Karenina principle^22^. We tested this hypothesis by comparing differences in beta diversity dispersion according to hatching status and fusariosis infection, using generalised least squares (GLS) models, which account for heterogeneity in variance between groups. We found no significant difference in fungal community dispersion driven by hatching or infection status (Supplementary Table 9b; null model AICc weight = 0.531; hatching status: *F*_*2,105*_ = 1.318; *p–value* = 0.272; infection status: *F*_*1*_,_*105*_ = 0.054; *p–value* = 0.817; Fig. 4h). Infection status was also not a significant predictor of bacteriome community dispersion (Supplementary Table 9a; adjR^2^ = 0%; *F*_*2,117*_ < 0.001; *p–value* = 0.989). In contrast, bacteriome beta diversity dispersion differed significantly according to hatching status (Supplementary Table S9a; ΔAIC = 47.84; adjR^2^ = 35%; *F*_*1,117*_ = 32.345; *p–value* < 0.001). The direction of the effect was opposite to that predicted by the Anna–Karenina principle, whereby hatched eggs had significantly higher beta diversity dispersion compared to failed E.D and L.D. eggs (glht Tukey comparisons: hatched eggs–failed E.D. eggs, *p–value* < 0.001; hatched eggs–failed L.D. eggs; *p–value* < 0.01; Fig. 4d). This result can be explained by the fact that failed unhatched eggs tended to have low microbial diversity compared to hatched eggs, resulting in higher among–individual variation in bacteriome composition in the diverse communities of healthy hatched eggs than in low–diversity unhatched eggs.

As the nest environment can influence the egg microbiome^20^, we repeated all beta diversity analyses, including the nest environment microbiome, yielding quantitatively equivalent results to those presented above (Supplementary Fig. 2). Although the nest is an important source of the inner egg microbiome, nest microbial communities remained distinct from the inner eggshell microbiome at all development stages. These results are consistent with our previous findings in the same population^20^ and are likely driven by host-mediated filtering processes. Notably, hatched eggs, which were opened by the emerging hatchling and thus more prone to environmental contamination, had a more distinct bacteriome from the nest environment than that of unhatched eggs, which were carefully dissected (Supplementary Fig. 2). If environmental contamination were driving the observed patterns, hatched eggs would be expected to closely resemble the nest environment, not less. This provides strong evidence that environmental contamination is not responsible for the observed patterns. In contrast, mycobiome composition of the eggshells was less distinct from the nest environment, likely reflecting the low diversity of fungal taxa present in the inner eggshells.

### Egg bacteriome and mycobiome diversity predict hatching success

We used GLMMs to test whether bacterial and fungal diversity and composition predict hatchability in interaction with infection status. To assess hatchability (i.e. the distinction between unhatched and hatched eggs), eggs that failed during the early or late stages of development were grouped and classified as unhatched eggs. We detected no significant effect of the interaction between infection status and any of the microbiome diversity metrics (Supplementary Table 10; p > 0.05), suggesting that microbiome and infection status effects on hatching are independent. Therefore, we excluded the interaction for all subsequent analyses.

**Fig. 5:**
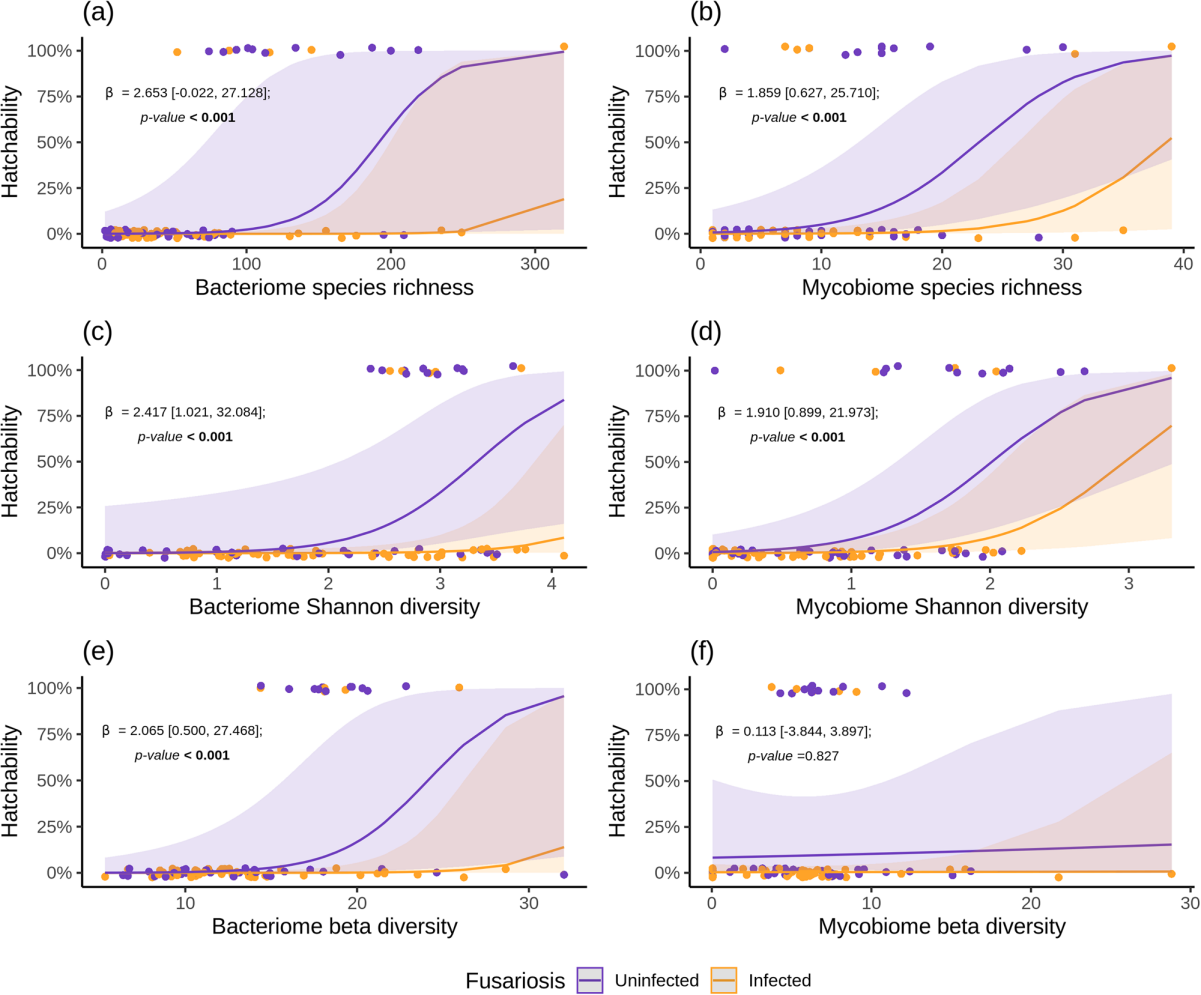
Bacteriome and mycobiome diversity in relation to hatchability. Bacteriome and mycobiome species richness (**a**, **b**), Shannon diversity (**c**, **d**), and beta diversity (**e**, **f**) in relation to hatchability. Fitted lines and 95% confidence intervals (shaded area) were estimated with a GLMM with a binomial distribution and nest ID as a random factor. For each model, fusariosis infection status was entered as an explanatory variable (FSSC uninfected in purple and FSSC infected in orange). Jitter is applied to the data points to show the repeated observations.

As anticipated and previously demonstrated^8^, fusariosis infection negatively predicted hatchability (Supplementary Table 11; p < 0.05). Additionally, all bacteriome and mycobiome alpha diversity metrics were significantly associated with hatchability (bacteriome species richness: *β* = 2.653; 95%CI [0.022, 27.128], df = 1; *χ*^2^ = 14.636; *p–*value < 0.001; Fig. 5a; mycobiome species richness: *β* = 1.859; 95%CI [0.627, 25.710], df = 1; *χ*^2^ = 13.746; *p–value* < 0.001; Fig. 5b; bacteriome Shannon diversity: *β* = 2.417; 95%CI [1.021, 32.084], df = 1; *χ*^2^ = 13.994; *p–value* < 0.001; Fig. 5c; mycobiome Shannon diversity: *β* = 1.910; 95%CI [0.899, 21.973], df = 1; *χ*^2^ = 16.218; *p–value* < 0.001; Fig. 5d). We observed that beta diversity dispersion of the bacteriome was also significantly associated with hatchability (*β* = 2.065; 95%CI [0.500, 27.468], df = 1; *χ*^2^ = 9.685; *p–value* = 0.002; Fig. 5e), suggesting that eggs with bacteriomes which were more distinct than the population average were more likely to hatch. This can be explained by the collinearity between Shannon diversity and beta diversity (Pearson correlation = 0.679; 95%CI [0.570, 0.765]; *t* = 10.092, df = 119, *p–value* < 0.001), with more diverse bacteriomes having higher beta diversity dispersion. Mycobiome beta diversity was not significantly associated with hatchability (*β* = 0.113; 95%CI [−3.844, 3.897], df = 1; *χ*^2^ = 0.048; *p–value* = 0.827; Fig. 5f). Overall, our findings show that greater microbiome diversity, of both bacterial and fungal taxa, is positively associated with hatching success independently of fusariosis infection, indicating that a diverse microbiome plays a functional and beneficial role in the egg, supporting embryonic development.

### Specific bacterial and fungal genera and their metabolic pathways are linked with fusariosis infection resistance and hatching success

**Fig. 6:**
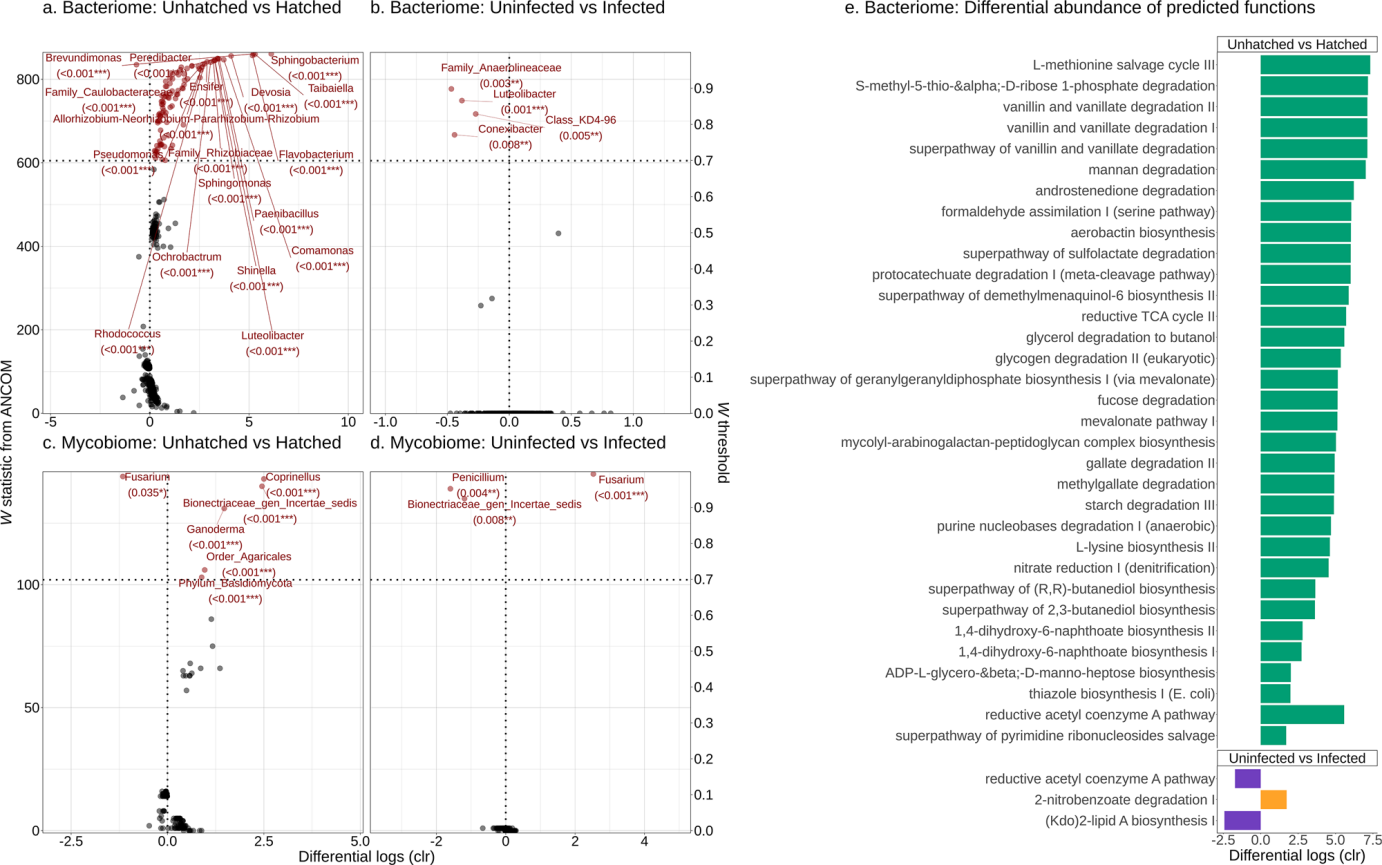
Differential abundance of specific bacterial, fungal taxa and bacterial predicted functional pathways according to hatching success and *Fusarium* infection status. Volcano plots of ANCOM analyses highlighting bacterial and fungal genera differing in abundance between (**a** and **c**) unhatched and hatched eggs, and (**b** and **d**) FSSC uninfected and infected eggs. Negative values on the x–axis indicate that a genus was more prevalent in uninfected (**b** and **d**) and unhatched (**a** and **c**) eggs. The values on the X–axis show the differential log estimates of centred logged ratios (CLR) for a given genus between the different groups according to a linear model. On the Y–axis, the *W* statistic indicates the number of instances in which the null hypothesis was rejected for a specific genus. The dots represent individual genera, with labelling applied when the *W* threshold was above 0.7, except in (**a**), where the genera were labelled when the *W* threshold was above 0.9. Adjusted *p*–values from the LMMs are added to the individual genera. **e** Barplot of differentially abundant (*W* < 0.7 and *p* < 0.05) predicted MetaCyc metabolic pathways of the bacteriome according to hatching and infection status based on the PICRUSt2 algorithm. For all analyses, the effect of hatching status was controlled for when testing for the effect of infection status.

To identify the specific bacterial and fungal genera involved in fusariosis infection resistance and hatchability, we employed an analysis of compositions of microbiomes (ANCOM)^23^. Comparing unhatched and hatched eggs, we identified 47 bacterial genera (ANCOM *W* > 0.7 and *p*–value < 0.05, Fig. 6a) and five fungal genera (ANCOM *W* > 0.7 and *p*–value < 0.05, Fig. 6c) that were positively associated with hatched eggs. These included the genus *Sphingobacterium* and a member genus of the family *Caulobacteraceae*, bacterial genera that have previously been reported to be part of a healthy inner egg microbiota in *P. unifilis*^20^. Higher relative abundance in hatched eggs was also found for the genera *Brevundimonas, Devosia, Ensifer, Sphingomonas, Paenibacillus, Peredibacter, Pseudomonas*, and a genus of the family Rhizobiaceae (Fig. 6a) and fungi from the genus *Bionectriaceae, Coprinellus* and *Ganoderma* and order *Agaricales* (Fig. 6c). When comparing uninfected and infected eggs, while controlling for the effect of hatching, we identified four bacterial genera (ANCOM *W* > 0.7 and *p*–value < 0.05, Fig. 6b) and two fungal genera (ANCOM *W* > 0.7 and *p*–value < 0.05, Fig. 6d) associated with uninfected eggs, including *Luteolibacter*, *Conexibacter*, one genus from the family Anaerolineaceae and one from the class *KD4–96* (Fig. 6b), and fungi from the genera *Penicillium* and family Bionectriaceae (Fig. 6d). In support of FSSC pathogens causing infection and contributing to hatching failure, we observed higher relative abundance of the fungal genus *Fusarium* in unhatched and infected eggs relative to hatched and uninfected eggs (Fig. 6c and d). We also report ANCOM comparisons between eggs that failed at early development (E.D.), late development (L.D.), and hatched eggs, as well as the effect of infection status within each group (Supplementary Figs. 3, 4, 5). Our results thus suggest that specific bacterial and fungal genera are associated with hatching success and fusariosis infection resistance.

We predicted MetaCyc metabolic pathways within the bacterial and fungal communities using the PICRUSt2 pipeline^24^ and estimated their relative abundance according to hatching and infection status using ANCOM^23^. None of the predicted mycobiome MetaCyc metabolic pathways were associated with hatching or infection status. However, predicting metabolic pathways from ITS data faces several limitations, including low prediction accuracy due to the limited number of reference genomes and associated gene–family annotations in mycobiome databases compared to prokaryotic databases^25^. Consequently, the current data do not allow us to infer the potential metabolic mechanisms underlying the observed associations between egg mycobiomes and hatching or infection status.

When comparing unhatched and hatched eggs, we identified 33 predicted bacterial MetaCyc pathways positively associated with hatched eggs (ANCOM W > 0.7 and *p*–value < 0.05; Fig. 6e.). These included pathways involved in aerobactin biosynthesis (AEROBACTINSYN–PWY), reductive acetyl coenzyme A (CODH–PWY), reductive TCA cycle II (PWY–5392), denitrification (DENITRIFICATION–PWY, PWY–6641), carbohydrate degradation (PWY–5941, PWY–6731, PWY–7456), aromatic compound degradation pathways (GALLATE–DEGRADATION–I–PWY, METHYLGALLATE–DEGRADATION–PWY, P184–PWY, PWY–7097/8), stress adaptation (PWY–1622), structural and signaling molecules (PWY–6397, PWY–922, PWY–5910), nucleotide and cofactor metabolism (PWY–6892, PWY0–1241) (Fig. 6e). When comparing uninfected and infected eggs while controlling for hatching status, we identified three predicted pathways (ANCOM W > 0.7): two positively associated with uninfected eggs and one with infected eggs. Specifically, the reductive acetyl–CoA pathway (CODH–PWY) and Kdo_2_–lipid A biosynthesis I (LIPASYN–PWY) showed higher relative abundance in uninfected eggs, whereas the 2–nitrobenzoate degradation I pathway (PWY–5647) was more abundant in infected eggs (Fig. 6e). These results suggest that shifts in the taxonomic composition associated with hatching and infection status are reflected in the functional profile of the bacteriome. In the context of egg fusariosis, the reductive acetyl–CoA pathway is of particular interest for future metagenomic and metabolomic studies, as it was enriched in both hatched and uninfected eggs (Fig. 6e).

### Hatchability is linked to the complexity of bacterial and fungal interkingdom networks

Microorganisms form complex interactions among community members^26^, and the degree of ecological associations can be used as an indicator of a healthy and functional host microbiome. Here, our objective was to investigate interkingdom associations in relation to fusariosis infection and hatching status. We constructed microbial association networks to identify co–occurrence interactions between the 23 most prevalent bacterial and 20 most prevalent fungal genera using Spearman correlations and netAnalyze from the NetCoMi package^27^. We compared positive and negative associations between bacteria and fungi found in unhatched (failed E.D. and L.D. eggs) and hatched eggs. Within these groups, we compared FSSC uninfected and infected eggs.

**Fig. 7:**
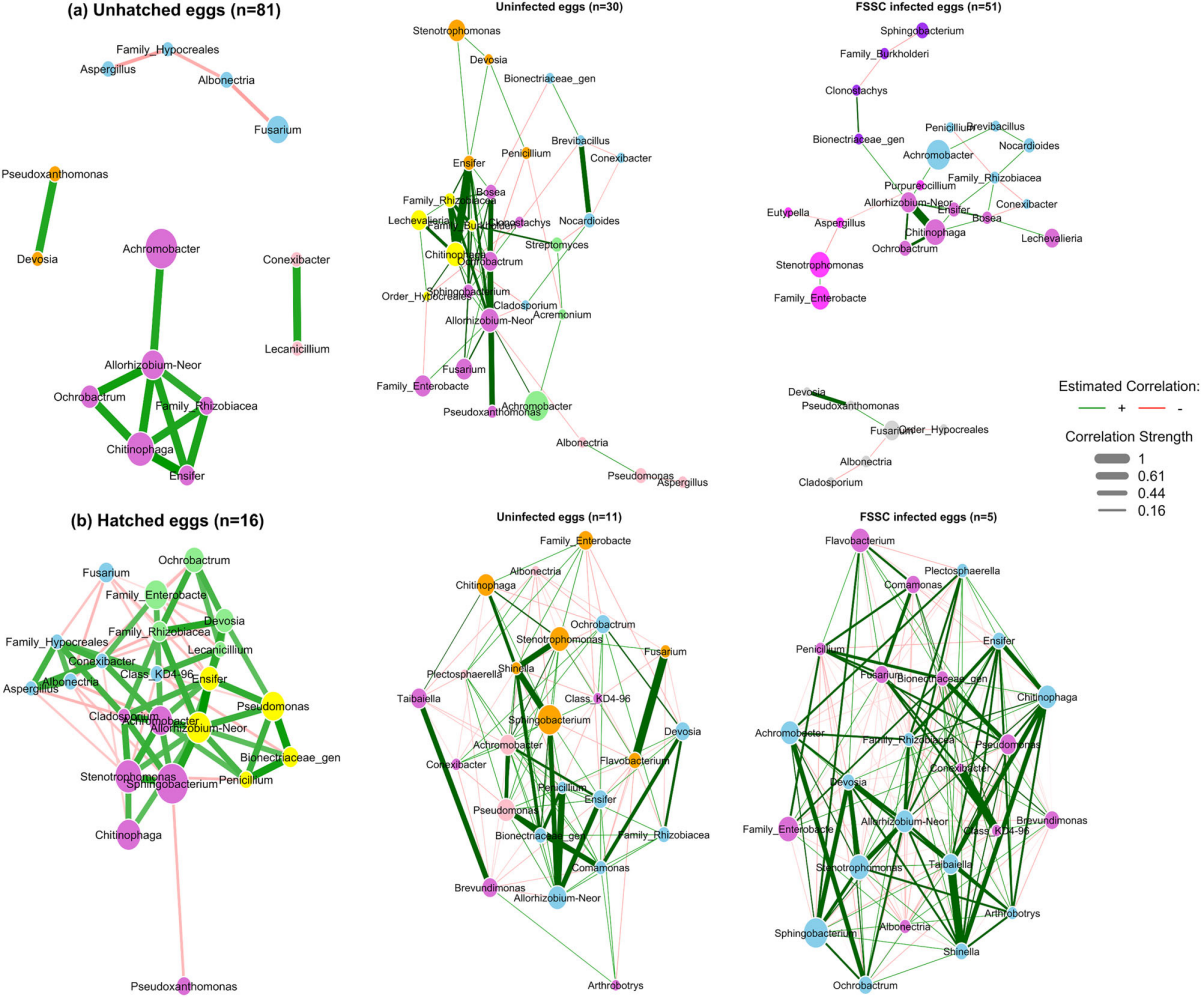
Microbial association networks to identify co–occurrence interactions between the 23 most prevalent bacterial and 20 most prevalent fungal genera in relation to FSSC infection and hatchability. The network analysis of (**a**) unhatched and (**b**) hatched eggs also illustrates the impact of FSSC infection. Networks were constructed using the Spearman method, with node size scaled to the sums of clr–transformed data and node colours representing the determined clusters. Single nodes were removed from the networks, with taxa labelled at the genus level. A green line indicates positive correlations between fungi and bacteria, while negative correlations are indicated by a red line. Thicker lines represent stronger Spearman correlations.

Unhatched eggs exhibited a simpler microbial network containing 14 nodes with 14 edges (density = 0.055) (Fig. 7a), while hatched eggs showed a more complex network containing 22 nodes with 71 edges (density = 0.281). Modularity was higher in hatched eggs (0.531 vs 0.214 in unhatched eggs), indicating intricate interactions between microbial members (Fig. 7b). Focusing only on uninfected eggs, hatched eggs displayed a more complex microbial network exhibiting 24 nodes with 116 edges (density = 0.617) compared to unhatched eggs showing 28 nodes with 62 edges (density = 0.125). Similarly, when infected eggs were considered, unhatched infected eggs had a less interactive network (27 nodes with 31 edges, density = 0.063), with the genus *Fusarium* forming part of a simple interconnected cluster. In contrast, in hatched infected eggs (24 nodes with 185 edges, density = 0.618), the genus *Fusarium* was a central node among many in a single and complex interconnected cluster, suggesting that infected eggs that hatch appear to maintain a functional microbial structure. Nonetheless, modularity in hatched infected eggs was lower (0.004) compared to hatched uninfected eggs (0.092), suggesting a disruption of the microbial community. These results highlight that hatching success is associated with a more complex and resilient microbial network and that, although *Fusarium* infection alters network dynamics, eggs that hatch despite infection appear to maintain a functional microbial structure.

## Discussion

Understanding the role of wildlife microbiomes in modulating resistance to emerging fungal infections is an urgent topic in biodiversity conservation. The global outbreaks of chytridiomycosis in amphibians and white–nose syndrome in bats have highlighted the crucial role of host–associated microbiomes in combating fungal diseases^11,28^. Our study in the yellow–spotted Amazon river turtle elucidates the role of the inner eggshell mycobiome in response to fusariosis infections and demonstrates that bacterial and fungal diversity of inner egg symbionts are associated with healthy embryonic development and hatching success.

A central paradigm of microbiome research is that greater diversity leads to increased stability and better protection against pathogen colonisation through competitive exclusion, as well as more efficient provision of services beneficial to the host, i.e. the diversity–stability hypothesis^29^. Consistent with this hypothesis, we observed that inner–egg mycobiome Shannon diversity was the strongest predictor of FSSC infection prevalence and intensity, with higher diversity associated with increased infection resistance. Similar results have been reported in the skin microbiome of hibernating bats in North America, where yeast diversity and abundance predicted white–nose Syndrome^16^. Our study is the first to establish a strong association between inner–egg mycobiome diversity and the likelihood of fusariosis fungal infection, demonstrating the importance of inner–egg mycobiome diversity in fungal disease resistance.

Recent studies suggest that egg microbiomes may be particularly critical for embryonic development in oviparous vertebrates that lack parental care, as parental transfer of beneficial symbionts cannot occur during incubation and rearing^30^. However, vertical transmission likely occurs during oviposition, when eggs are coated with the mucosa from the maternal cloaca. Indeed, a recent study in the Chinese three–keeled pond turtle (*Mauremys reevesii*) showed that freshly laid eggs had significant similarities with the cloacal microbiome of the laying turtle^18^. Yet how this initial inoculum shapes the outer and inner eggshell microbiome in interaction with environmental nest microbiomes throughout development remains to be elucidated.

We found that eggs that failed to develop at an early embryonic stage exhibited significantly lower bacterial diversity than those that failed at a later stage, suggesting that diversity increases with development. A possible mechanism is calcium resorption during embryonic development, which results in eggshell thinning and increased permeability to microorganisms from the outer eggshell and the nest environment^31–33^. Moreover, hatched eggs had higher bacterial and fungal diversity than eggs that failed to hatch, indicating that increased bacteriome diversity during later stages of development is a marker of healthy embryogenesis. Our findings align with studies in fish demonstrating that increased bacteriome and mycobiome diversity of eggs correlates with longer developmental times^34,35^.

In addition to diversity, we observed strong differences in bacterial composition between hatched eggs and eggs that failed at early and late developmental stages. Hatched eggs had significantly higher dispersion (i.e. variability) in bacterial composition compared to failed eggs. The anti–Anna Karenina principle for microbiomes offers an insightful explanation for this pattern^22^: when microbiomes are disrupted, diversity declines, giving rise to more homogeneous communities^22^, and consequently variation in composition between individuals will be greater among diverse communities than among those of low diversity. Consistent with this prediction, we found that increased species diversity was correlated with greater beta diversity dispersion. Thus, eggs that hatched were associated with more diverse and compositionally variable microbial communities than eggs that failed, providing convincing evidence that a diverse egg microbiome is essential for healthy embryogenesis.

Given the robust differences in microbial diversity and composition identified during embryogenesis, we assessed the impact of microbiome diversity on hatching success. We found a strong association between bacterial and fungal species richness and evenness and hatchability, which remained robust even when accounting for fusariosis infection. Dispersion in bacterial composition was a significant predictor of hatchability, consistent with the anti–Anna Karenina principle outlined above. These results further highlight that eggs with diverse and complex inner–egg microbiomes are more likely to hatch successfully. In fish eggs, higher bacterial and fungal diversity has been shown to correlate with improved hatching success^34,35^. Our results in the yellow–spotted Amazon river turtle extend these findings, demonstrating that inner–egg microbiome diversity is a strong predictor of hatching success regardless of fungal infection.

The egg–associated microbiome may also provide protective properties against pathogens, acting as the first line of defence against fungal infections. Our results showed no direct effect of fusariosis infection on microbiome diversity throughout development. However, fusariosis infection significantly impacted the composition of the microbiome. Specifically, there were significant differences in bacterial communities between uninfected and FSSC–infected eggs that failed at late development, suggesting that infection alters microbial composition at late embryonic stages. Interestingly, we also observed significant differences between infected eggs that failed at late development and infected eggs that successfully hatched. This suggests that eggs at a late embryonic development, which are able to hatch despite infection, may harbour beneficial bacterial symbionts that help mitigate the negative effects of fungal infection. Indeed, an experimental study in the striped plateau lizard (*Sceloporus virgatus*) demonstrated that egg bacterial communities can play an essential role in fungal disease suppression and contribute to hatching success^17^. Furthermore, we found that fusariosis infection significantly impacts mycobiome composition, with FSSC pathogens dominating in infected eggs. This suggests that successful colonisation of the inner egg by FSSC pathogens results in their overgrowth within the already limited symbiotic fungal community, leading to a change in the fungal community structure of the inner egg. Overall, our results align with existing evidence that the presence of defensive symbionts is an evolutionary mechanism ensuring healthy development^30,36^, and further suggest that beneficial symbionts may be vertically transmitted, a question that remains unresolved for oviparous vertebrates lacking parental care.

Currently, there is a lack of studies in oviparous vertebrates reporting microorganisms that confer fungal disease suppression, particularly in eggs. Here, we report specific bacterial and fungal genera and predicted bacterial metabolic pathways within the inner eggshell associated with fusariosis infection status. Uninfected eggs had a higher abundance of bacteria from the family Anaerolineaceae and the genus *Luteolibacter*, which have been linked with fungal disease suppression and observed to increase in abundance in the rhizosphere following *F. oxysporum* infection^37,38^, as well as bacteria from the class KD4–96, known to be key bioindicators of high biological health status in soils^39^. A higher relative abundance of the predicted reductive acetyl–CoA and the Kdo2–lipid A biosynthesis pathways was also found in uninfected eggs. The former is involved in the stoichiometric conversion of glucose to acetate and in carbon dioxide fixation, using hydrogen as an electron donor to produce acetyl–CoA–a central molecule in energy metabolism and biosynthesis^40^. In the human gut, bacterial–derived acetate plays a key role in immunomodulatory mechanisms that control inflammation and protect the host from pathogen invasion^41^. Kdo2–lipid A is essential for bacterial survival and is a key molecule in host–pathogen interactions, particularly in triggering immune responses^42^. These findings suggest that both pathways may contribute to resistance against egg fusariosis; however, further experimental validation using metagenomic and metabolomic approaches is needed to test this hypothesis. Interestingly, the predicted 2–nitrobenzoate degradation I pathway was more abundant in FSSC–infected eggs. This pathway enables bacteria to degrade nitroaromatic compounds using carbon as an energy source, and a high relative abundance may indicate environmental stress or toxicity^43^, which could negatively impact embryo development and hatching. Although we found no effect of infection status on predicted fungal metabolic pathways, we observed a higher relative abundance of the fungal family Bionectriaceae in uninfected eggs. Members of this family are known for producing secondary metabolites with cytotoxic activities and compounds that can neutralise antifungal metabolites, including those produced by *Fusarium spp*.^44,45^. Similarly, *Penicillium* was found in higher abundance in uninfected eggs and is well known for its antibacterial and antifungal properties^46^. Thus, both Bionectriaceae and *Penicillium* may play an important role in suppressing fusariosis infections. Whilst further experimental and culture–based studies are needed to elucidate the underlying mechanisms, our results are consistent with the hypothesis that inner eggshell–associated bacteria and fungi contribute to the protection against fungal infections during the incubation period.

We also found specific genera linked with hatching success. Hatched eggs had a higher relative abundance of bacteria belonging to the genera *Brevundimonas*, *Ensifer*, *Flavobacterium*, *Sphingobacterium*, *Sphingomonas*, *Pseudomonas*, and the family Rhizobiaceae. All these taxa have previously been reported as part of the normal egg microbial composition of oviparous vertebrates, including this turtle species^20^, birds^47,48^ and frogs^49^. Bacteria from the genus *Rhizobium* can directly counteract fungal pathogens by producing antibiotics and enzymes that degrade fungal cell walls. Similarly, *Ensifer* uses mycolytic enzymes to suppress the fungal pathogen *Fusarium oxysporum* and enhance growth in crops^50^. Bacteria from the genera *Paenibacillus* and *Peredibacter* were also found in higher abundance; the former is well known for protecting crops against pathogens, including bacteria, fungi and viruses^51^ and the latter for its predatory behaviour against other bacteria^52^. We found several predicted metabolic pathways associated with energy and nutrient supply to be more abundant in hatched eggs, including the reductive acetyl–CoA pathway^40,53^. Pathways related to iron competition and immune modulation were also enriched. For example, aerobactin biosynthesis limits iron availability to pathogens, while ADP–L–glycero–β–D–manno–heptose biosynthesis may stimulate host immunity, contribute to bacterial cell wall structure, and influence immune recognition^54,55^. Collectively, the predicted functional pathways in hatched eggs suggest that bacterial symbionts may create an optimal biochemical environment – supporting embryonic development, modulating host immunity, and potentially suppressing pathogens. We acknowledge the limitations of predicting microbial function based solely on taxonomic data^25,56,57^. Nonetheless, our findings provide a valuable foundation for future studies that integrate experimental infection models with metagenomic and metabolomic analyses.

In hatched eggs, the fungal genera *Coprinellus* and *Ganoderma*, along with members of the family Bionectriaceae, were present at notably higher relative abundances. Currently, there are no reports of these fungi in the eggs of oviparous species, preventing the assessment of their functional roles or potential influence on hatching success. Fungi from the genera *Coprinellus* and *Ganoderma* are typically saprotrophic and commonly found in decaying wood or dung^58,59^. Therefore, their presence in the inner eggshells may result from spore contamination. In support of this, these taxa were almost exclusively detected in hatched eggs (see Supplementary Fig. 6), suggesting that contamination may occur during or after hatching. However, *Coprinellus* species are also hyphae–forming fungi and have recently been implicated in human keratitis^60^, indicating their capacity to exhibit both biotrophic and pathogenic lifestyles. Thus, we cannot exclude the possibility that *Coprinellus* may act as a commensal or even beneficial symbiont in reptilian eggs.

In contrast, Bionectriaceae was prevalent in eggs across all developmental stages (see Supplementary Fig. 6), but at higher relative abundance in hatched eggs. As noted above, taxa from this family produce secondary metabolites with cytotoxic activity, enabling them to act as mycoparasites—a trait that has been demonstrated specifically against mycotoxigenic *Fusarium* species^44,45^. Although little is currently known about the specific roles of bacteria and fungi in hatching success, our findings strongly suggest that certain microbial taxa are associated with successful hatching and may offer protective functions, such as suppressing fungal infections and interacting with the embryo’s immune system. This is consistent with studies showing that egg–associated microbial communities can defend against pathogens by producing antimicrobial compounds that protect developing embryos^30^. Such defences are likely to be particularly important in oviparous vertebrates that lay eggs in environments rich in microorganisms, such as marine and freshwater habitats^61,62^, and specifically in turtles, where FSSC pathogens may be present in the nesting environment^4^. Further research is needed to elucidate the mechanisms underlying these interactions.

Finally, following the hypothesis that complex microbial interactions between bacteria and fungi are indicators of a functional microbiome^63^, we tested whether differences in network interactions are linked with hatchability and infection resistance. We observed considerably more complex interkingdom interactions in hatched eggs compared to unhatched eggs. Eggs that hatched despite fungal infection maintained the complexity of interactions and exhibited a functional microbial structure. In hatched eggs, we observed increased abundances of numerous bacterial and fungal taxa, among which Bionectriaceae, *Pseudomonas*, *Sphingobacterium, Ensifer*, *class KD4–96*, and Rhizobiaceae were positively correlated with one another and negatively correlated with the genus *Fusarium*. Consistent with our results, complex microbial network interactions and higher abundance of symbiotic bacteria in corals have been associated with pathogen–free environments less impacted by organic pollution^64^. Similarly, bacteria and fungi associated with maize stalks showed negative correlations with *Fusarium* pathogens^65^. Network interactions reflect the complexity of ecological relationships among microorganisms; the more stable and diverse a microbiome, the more complex its interactions tend to be, which can reduce susceptibility to microbial invasions^29,66^. Thus, we show that complex interkingdom microbial interactions within the egg–associated microbiota are associated with higher hatching success and are key for successful hatching despite fusariosis infection.

In conclusion, our study reveals the pivotal role of the inner egg bacteriome and mycobiome diversity in fusariosis disease suppression and hatching success. We acknowledge that these results are based on 16S and ITS metabarcoding, which have limitations in identifying specific microbial functions^25,56,57^. Moreover, understanding the causal mechanisms underlying microbiome–pathogen interactions in eggs will require longitudinal and experimental approaches capable of disentangling the direct effects of infection on microbiome composition from host–mediated responses^67^. Nonetheless, our study identifies key putative microbial taxa and predicted functional pathways potentially involved in fusariosis suppression and hatching success. Crucially, the bacteria and fungi identified in this study have previously been found to be associated with the suppression of *Fusarium spp*. pathogens in other systems and form part of the healthy egg microbiota in other oviparous vertebrates. Our results suggest that microbial richness is associated with more complex interkingdom interactions, which may support healthy embryonic development and contribute to disease suppression. These findings provide valuable insights for future fungal pathogen research and the development of effective conservation strategies involving probiotics. Further research is needed to identify key microbial species and their antifungal compounds, with the long–term goal of developing conservation practices, such as treating artificial nesting sites with host–associated microbial symbionts, to help reduce or stabilise fungal pathogen growth within nests.

## Methods

### Study site and sample collection

The yellow-spotted Amazon freshwater turtle (*Podocnemis unifilis*) is found throughout the northern Amazon region, the Orinoco, Amazon and Essequibo river basins, and the eastern Guianas. This turtle species is threatened by over-harvesting in much of its geographic range. Since 2007, a reintroduction project for the Yellow-spotted Amazon River Turtle has been established at the Tiputini Biodiversity Station (TBS) from the Universidad San Francisco de Quito in the Yasuni Biosphere Reserve, Orellana Province, 280 km ESE of Quito, Ecuador (0°38′18″S 76°9′0″W). To stabilise and maintain the population of this freshwater turtle, around 700 eggs are collected each year from various nests along the banks of the Tiputini River and hatched in captivity in artificial nests. The artificial nests are filled with sand from the nesting beaches, where the clutches are collected, to maintain the natural conditions of the nest as closely as possible. The turtles are kept in artificial ponds filled with river water for one to two months to ensure their successful release back into their natural habitat, the Tiputini River.

We collaborated with the TBS Conservation Management Programme for our study and used the yellow–spotted Amazon river turtle as our model organism to investigate whether the microbiota present on the inner eggshells could predict the risk of fusariosis infection and hatching success. We have complied with all relevant ethical regulations for animal use. A total of 127 inner egg swab samples were collected from 31 artificial nests. The developmental status of eggs classified into three developmental categories: 1) eggs that failed at an early developmental stage (less than 30 days of incubation, termed failed early development, E.D.), 2) eggs that failed to hatch after 100–120 days of incubation with a viable developed embryo at a late developmental stage (termed failed late development, L.D.), and 3) successfully hatched eggs (Fig. 1, Supplementary Table 1). All sampled unhatched eggs, E.D. and L.D., were intact before sampling, with no fractures or signs of having been opened. All the eggs showing a sign of being open or having a fissure were not considered for the study to avoid sampling of saprophytic bacteria. Hatched eggs were collected immediately after the hatchling had entirely emerged to minimise environmental contamination from the nest. Swabs from the sand of the artificial nests were also collected to control for contamination and the influence of the nest microbiome on the egg microbiome (see^20^).

Additionally, we aimed to determine whether the eggs were infected by the three primary FSSC pathogens—*Fusarium solani, F. keratoplasticum*, and *F. falciforme—*known to cause fusariosis in turtle eggs. During fieldwork, eggs were classified as symptomatic for fusariosis when covered with unusually coloured spots (green, pink, greyish) and having a non–uniform shape; eggs without these signs were classified as asymptomatic. All egg samples were subsequently tested for fusariosis infection using *Fusarium–*specific primers from the TEF1 alpha gene as described in ref. ^8^. Inner eggshell samples were collected by swabbing the inner shell surface for up to 5 s. After collection, each swab sample was placed in a 1.5 ml Eppendorf tube containing Nucleic Acid Preservation (NAP) buffer. To control for environmental contamination from the ambient air, field blanks were collected by swabbing the air for one minute.

### Assessment of inner egg bacteriome, mycobiome and FSSC pathogens using Illumina high–throughput sequencing

DNA was extracted from swab samples using the NucleoSpin^®^ 96 Soil extraction kit (Macherey–Nagel, Germany). Samples were eluted with 70 μl of preheated SE buffer, followed by a two–step PCR for all samples. In the first step, we targeted the V4 region of the bacterial 16S rRNA gene using primers 515 F (5’–GTGCCAGCMGCCGCGGGTAA–3’) and 806 R (5’–GGACTACHVGGTWTCTAAT–3’)^68^ to characterise the bacteriome; the internal transcribed spacer (ITS) region using primers ITS1F (5’–CTTGGTCATTTAGAGGAAGTAA–3’) and ITS4R (5’–TCCTCCGCTTATTGATATGC–3‘)^69,70^ to characterise the mycobiome; and a 430 bp region of the translation elongation factor 1–alpha gene (TEF1 alpha) using primers Fa_150 (5’–CCGGTCACTTGATCTACCAG–3’) and Ra–2 (5’–ATGACGGTGACATAGTAGCG–3’)^71^ to test for the presence of *Fusarium* species causing fusariosis. All primer pairs were extended with universal sequences CS1 (ACACTGACGACATGGTTCTACA) + 4 N and CS2 (TACGGTAGCAGAG–ACTTGGTCT) for forward and reverse primers, respectively (Standard Biotools, USA), which served as adapters in the second PCR step.

For the first–step PCR, a total volume of 10 µl was used. Each reaction received 5 µl Amplitaq Gold 360^™^ mastermix (Applied Biosystems, USA), 2.5 µl purified water, 3 µl (300 nM) primer mix, and 1 µl template DNA. The amplification of the 16S rRNA V4 region involved an initial denaturation at 95 °C for 10 min, followed by 30 cycles of denaturation at 95 °C for 30 s, annealing at 60 °C for 30 s, and extension at 72 °C for 45 s, ending with a final extension at 72 °C for 10 min. For the ITS region, the conditions were similar, with an annealing temperature of 54 °C and an extension time of 60 s. The TEF–alpha gene was amplified with 35 cycles at an annealing temperature of 52 °C. Gel electrophoresis was conducted to confirm PCR success.

The second–step PCR was performed in 20 µl reactions in 96–well plates to add Illumina sequencing adapters and unique barcodes. A master mix containing 10 µl AmpliTaq Gold™ 360 mastermix(Applied Biosystems, USA) and 3.5 µl ultrapure dH_2_O was prepared. Each well was loaded with 13.5 µl mastermix, 4 µl of a unique barcode (Acces Array™ Barcode Library for Illumina Sequencers-384, Single Direction, Standard Biotools, USA) and 2.5 µl of the first step PCR amplification used as an input template; this process was done for each of the markers used in this study. Barcoded samples were purified using the NucleoMag^®^ NGS Clean–Up and Size Select Kit (Macherey–Nagel, Germany) with a 1:1 ratio of amplicon to beads on a Gene Theatre (Analytik Jena, Germany) following the manufacturer’s instructions. The purity of the amplicons was controlled in a QIAxcel Advanced System (QIAGEN, Germany). The clean amplicons were then quantified with the Quantifluor® dsDNA System (Promega Corporation, USA, Madison) on a TECAN Infinite F200 Pro (Tecan Trading AG, Switzerland) following the manufacturer’s instructions. Clean amplicons were eluted to 20 µl quantified (QuantiFluor^®^ dsDNA System, Promega, USA), pooled in equimolar amounts of 12 ng DNA per sample, and further diluted the pool to 6 nM. The library was prepared according to Illumina protocol and then sequenced at 12 pM on an Illumina MiSeq instrument using a 250 bp paired–end strategy, including 10% PhiX to account for low diversity. Three negative field controls, six negative DNA extraction controls, and nine negative PCR controls were included, referred to as ‘blanks’.

### Bioinformatic processing of the bacteriome

All paired–end sequencing reads from 127 successfully sequenced inner egg swab samples for the 16S rRNA gene V4 region were pre–processed using the QIIME2^72^ microbiome analysis pipeline (version 2019.1) and DADA2^73^ to denoise the data and generate amplicon sequence variants (ASVs). We assigned taxonomy to the resulting 14 191 ASVs using the Silva (version 132) V4^74^ classifier as a reference and removed sequences classified as chloroplast, mitochondria, archaea, Eukaryote and unclassified phylum, ending with 13 938 ASVs. Using MAFFT^75^, we added an archaeal sequence to root a tree and constructed a phylogenetic tree using FastTree 2.1.8^76^. After denoising, assigning taxonomic and filtering contaminants, we obtained 7’292,052 reads with an average of 56,969.15 per sample for downstream analyses. Contaminating bacterial DNA is commonly found in different buffers and extraction kits^77^, and contaminants appear at higher frequencies in PCR controls than in positive samples^78^. Therefore, we aimed to remove ASVs found in 18 successfully sequenced blanks (3 field blanks, six extraction blanks and 9 PCR blanks). We imported the data generated by QIIME2 using the R package“phyloseq”^79^. In R^80^ we used the function *decontam::isContaminant*^78^ using the “prevalence” method to identify and remove the blank microbiome from the dataset. This filtering step resulted in the removal of 26 ASVs in the dataset. Finally, samples with fewer than 10,000 reads were also excluded, removing six inner egg samples from our initial dataset. This left us with a final dataset that totals 6’587,609 reads across 13,912 ASVs and 121 samples, yielding an average of 54,443.05 reads per sample. Preliminary rarefaction curve analysis indicated that ASV richness plateaued at sequencing depths of 10,000 reads and above (Supplementary Fig.7a). Sequencing depth was not a significant predictor of bacteriome alpha diversity (species richness: df = 1, *χ*2 = 1.526, *p*–value = 0.217; Shannon diversity: df = 1, *χ*2 = 3.530, *p*–value = 0.060). As all samples exceeded this threshold, we retained the full dataset without rarefying to maximize detection of rare taxa.

### Bioinformatic processing of the mycobiome

A total of 114 successfully sequenced samples for the ITS region were subjected to pre–processing. The QIIME2^72^ microbiome analysis pipeline (version 2019.1) and DADA2^73^ in R were employed to denoise the dataset and generate amplicon sequence variants (ASVs). Because the ITS region varies substantially in length (700–900 bp), which complicates the standard DADA2 filtering and trimming workflow in QIIME2, we pre–processed the sequences in three steps.

We began by using the cutadapt^81^ plugin of QIIME2 to remove primers. Next, we extracted the ITS1 forward reads and ITS2 reverse reads (which encompass the two ITS regions) from the.qzv files and saved them into.fastq files. These files were placed in a new folder for further processing. Following the DADA2 ITS pipeline workflow (73; https://benjjneb.github.io/dada2/ITS_workflow.html), the ITS1 forward and ITS2 reverse reads without primers were quality-filtered using *filterAndTrim()* with maxEE = c^5^, maxN = 0, and truncQ=2. Error rates were learned separately for forward and reverse reads using *learnErrors()*, and sample inference was performed with *dada()*. Since the ITS amplicon length exceeded the capacity for paired-end read overlap, forward and reverse reads were joined using *mergePairs()* with *justConcatenate = TRUE*. An ASV table was constructed with *makeSequenceTable()*, and chimeric sequences were identified and removed using *removeBimeraDenovo()* with method =“consensus”. A.fna file was created to export the representative sequences to QIIME2 for taxonomic assignment.

We used the UNITE database release 9.0 2022^82^ for taxonomic assignment. Before building the classifier, we de-replicated the database using the dereplicate plugin of RESCRIPt^83^ in QIIME2 and compared different approaches of dereplication and filtering, yielding up to 54.09% of species coverage. Dereplication is recommended to eliminate redundant sequence data from databases. Maintaining redundancy in a reference database causes sequencing reads to be either randomly distributed across redundant sequences, resulting in a single random alignment reported from many possible options, or to be reported at all redundant locations. This redundancy skews the analysis of relative abundance, particularly for fungal diversity across samples, making it appear that multiple ecologically equivalent populations coexist and leading to an artificially low estimate of each taxon’s relative abundance^84^. After de-replication, a classifier was built, and taxonomy was assigned to 6453 ASVs.A phylogenetic tree was inferred in R using the phangorn package^85^. Representative sequences were aligned using DECIPHER’s *AlignSeqs()* function. A neighbor-joining starting tree was estimated from maximum-likelihood distances, then optimized under a GTR + Γ+I model using *optim.pml()* with stochastic tree rearrangements. The tree was rooted using midpoint rooting.

After denoising, taxonomic assignment, and contaminant filtering, we obtained 2,025,198 reads across 5200 ASVs in 114 samples, with an average of 18,410.89 reads per sample for downstream analyses. Following the same approach as for the bacterial microbiome, we removed ASVs found in 18 successfully sequenced blanks (3 field blanks, 6 extraction blanks, and 9 PCR blanks) using *decontam::isContaminant*^78^ with the“prevalence” method. This filtering step resulted in the removal of 3 ASVs. Finally, we excluded samples with fewer than 3,000 reads, resulting in a final dataset of 2,013,391 reads across 5,197 ASVs and 109 samples, with an average of 18,471.48 reads per sample. Preliminary rarefaction curve analysis indicated that ASV richness plateaued at sequencing depths of 3,500 reads and above (Supplementary Fig. 7b). Sequencing depth was not a significant predictor of mycobiome alpha diversity (species richness: df = 1, *χ*2 = 1.155, *p*–value = 0.283; Shannon diversity: df = 1, *χ*2 = 0.590, *p*–value = 0.442). As all samples exceeded this threshold, we retained the full dataset without rarefying to maximize detection of rare taxa.

### Taxonomic assignment of *Fusarium* (FSSC) pathogens

All paired-end sequencing reads from 111 successfully sequenced inner egg swab samples were pre-processed as described for the 16S and ITS markers. Forward and reverse read pairs were trimmed using cutadapt^81^ plugin of QIIME2 and filtered using DADA2^73^, with forward reads truncated at 225 nt and reverse reads at 225 nt. No ambiguous bases were allowed, while each read was required to have less than two expected errors based on their quality scores. To assign taxonomy to 188 ASVs from the TEF1-alpha gene, we constructed a database from the NCBI Genbank using RESCRIPt^83^ via QIIME2. We used the query“*elongation factor 1-alpha[All Fields] AND fungi[filter*]” to filter the database sequences by a minimum length of 100 and built a classifier^8^. Before using the classifier for taxonomy assignment, we trained the databases with the specific TEF primers used in this study. Only sequences assigned to *Fusarium* or the teleomorph names (*Gibberella and Nectria*) and longer than 300 bp were retained for further analysis. After denoising, assigning taxonomic and filtering contaminants, we obtained 971,153 reads across 123 ASVs and 104 samples, with an average of 9,338 reads per sample for downstream analyses. For this study, we only took the information of the samples with an FSSC (*Fusarium solani and Fusarium keratoplasticum*) ASV count greater than one and added it as a variable to the overall bacteriome and mycobiome metadata, which resulted in a total of 650,837 reads across 64 ASVs and 72 samples. After the bacteriome pre processing only 63 FSSC infected samples remained for further analyses.

### Statistical analyses in the R environment

Analyses were carried out in R version 4.3.3. We used the R package phyloseq^79^ to import the data generated by QIIME2 and to estimate alpha diversity (within samples) and beta diversity (between samples). For both 16S and ITS markers, we estimated two measures of alpha diversity: species richness, representing the total number of ASVs recorded, and the Shannon index, which accounts for ASV abundance.

For beta diversity estimation, treating microbiome data as compositional data is increasingly recognized^21,86,87^. Therefore, we applied the robust Aitchison log–ratio approach, which consists of transforming the data using the robust centred log–ratio (rCLR) transformation via the *microbiome::transform* function. The rCLR transformation computes the geometric mean from non–zero values only, thereby avoiding pseudocount–induced bias in sparse datasets^86^. Before beta diversity analysis, ASVs with a phylogenetic distance of less than 0.05 nucleotide substitutions per site were collapsed using *tip_glom()* function (*h* = 0.05) in phyloseq. Redundancy analyses (RDAs) were performed using the rda function from the R package vegan^88^ to assess the effects of egg developmental or infection status on community composition. Pairwise comparisons were performed using the *multiconstrained* function of the R package BiodiversityR^89^. We conducted a principal component analysis (PCA) to visualise beta diversity distances between groups.

To investigate differences in the relative abundance of ASVs according to hatching and infection status, we used analysis of the composition of microbiomes (ANCOM)^23^. ASVs were agglomerated at the genus level using the *phyloseq::tax_glom* function. ANCOM generates *W* scores, where higher *W* scores indicate stronger evidence that the relative abundance of a given genus is associated with a given predictor^23^. We used volcano plots to visualize the relationship between the *W* score and the differential log estimates from a linear model. A *W* score threshold of 0.7 and a *P* value of < 0.05 from linear models was used to identify taxa or predicted pathways whose relative abundance differed according to infection or hatching status. Because hatching status had a strong effect on microbiome composition, we controlled for its effect when testing for the effect of infection status. We used the PICRUSt2 pipeline^24^ to obtain the predicted MetaCyc metabolic pathways for the bacteriome using a min–alignment of 0.8. For the mycobiome we used the PICRUSt2 pipeline through FROGSFUNC^90^ because of its capability to perform this analysis on ITS data. The min–alignment used was 0.6. The abundance of predicted MetaCyc pathways was analysed using ANCOM following the same criteria as described above for taxonomic analyses.

To investigate potential interactions between microbial taxa we performed network analysis considering significant taxon co–occurrence patterns using the package NetCoMi^27^. Because network associations strongly depend on microbial abundance or sequencing depth (e.g. if the microbial load is high, both taxon 1 and taxon 2 will have high abundances even if they are negatively associated, leading to false positive associations), we accounted for the overall bacterial load. The data was transformed using the rCLR transformation with the *microbiome::transform* function. As done for the ANCOM analysis, we agglomerated ASVs at the genus level using the *phyloseq::tax_glom* function. We built networks according to hatchability (unhatched and hatched eggs) and infection status within each hatchability group (uninfected vs FSSC infected) using the NetCoMi package^27^.

### Statistical methodology for each research question

#### Does the diversity of fungal community in turtle eggs confer resistance to fusariosis infection prevalence and intensity?

We used generalized linear mixed models using the quantified inner eggshell bacterial and fungal diversity (species richness and Shannon index) and community dispersion as the response traits, to account for the non-independence of sampling multiple eggs from the same nest (pseudo-replication), we included NestID as a random effect in all the models to investigate potential associations between *Fusarium* prevalence and intensity and bacterial and fungal diversity. As each egg was sampled only once, we did not include EggID as a random effect.

We first tested whether bacterial and fungal diversity and community dispersion can predict FSSC infection in the three egg development stages (failed E.D., failed L.D., and hatched eggs) and whether these microbiome traits increase/enhance hatching success. The response variables were FSSC infection status, where a binomial matrix, 0 = negative *Fusarium-*PCR test, 1 = FSSC infection determined by TEF alpha gene target sequencing, was created to test FSSC prevalence; and FSSC ASV abundance (zero-truncated ASV counts of *F. solani* and *F. keratoplasticum* strains) to test FSSC infection intensity. Using generalised mixed models (GLMMs) with a negative binomial distribution, we tested whether bacterial and fungal species richness, Shannon diversity, and community dispersion predicted fusariosis infection prevalence. Using zero-truncated GLMMS with a truncated negative binomial distribution, we tested whether bacterial and fungal species richness, Shannon diversity, and community dispersion predicted the intensity of FSSC infection. We presented separate plots for each diversity measure modelled with the response variable. For the binomial data, jitter was applied to the data points to show sample size.

#### Is the healthy embryonic development linked to microbial diversity and composition?

We investigated whether the bacterial and fungal diversity and community structure varied between eggs at the three developmental stages (failed E.D. eggs, failed L.D. eggs, and hatched eggs) and eggs with different infection statuses (uninfected and FSSC-infected eggs).

First, we assessed alpha diversity (species richness and Shannon index) and performed GLMs with a negative binomial distribution and GLMs with a gamma distribution, respectively, using the R package“mgcv”^91^. In the full models, we included the covariates developmental status (failed E.D., failed L.D., and hatched eggs) and infection status (FSSC uninfected and FSSC infected). Additionally, we performed Tukey’s test from our model to test the differences between pairs within the groups and reported the p-values in the text and the plots.

Second, we tested whether bacteriome and mycobiome composition is independently driven by developmental status and FSSC infection status or by the interaction of the two variables. We performed permutation tests using RDA’s in three separate models, one with the variable developmental status (failed E.D., failed L.D., and hatched eggs), a second with the variable FSSC infection status (FSSC uninfected and FSSC infected) and a third with the interaction between the developmental status*FSSC infection status, each model with 99,999 permutations. We presented each of the results in different PCAs.

Lastly, we investigated whether the variation in bacteriome and mycobiome composition is associated with egg developmental status or FSSC infection status. We predicted that heterogeneity of beta diversity (dispersion from the population median) increases as the embryo develops in the egg since, during incubation, the nest microorganisms will colonise the internal egg. As time passes, the contribution will be more significant, as shown previously in this species^20^. Moreover, we predicted that the heterogeneity of beta dispersion would increase in FSSC-infected eggs. To test these predictions, we used the *betadisper* function with the R “vegan” package using Euclidean distances and the median distance to the centroid within developmental and infection status. We performed GLS to control for microbial composition heterogeneity during egg developmental status and infection status using the R package“nlme”^92^. In the full model, we entered the covariate developmental status (failed E.D., failed L.D., and hatched eggs) and the covariate fusariosis infection status (FSSC uninfected and FSSC infected). As stated above, we performed Tukey’s test from the GLS models to test the differences between the pairs of the two groups (developmental status and infection status).

#### Do egg bacteriome and mycobiome diversity predict hatching success?

As explained above, we constructed logistic regression models using the quantified inner eggshell bacterial and fungal diversity (species richness and Shannon index) and community dispersion as the response traits predicting the probability of hatching success. We included the nest ID as a random factor in all the models to control for group effect. We used generalised mixed models (GLM) with the binomial family. In the full models, we included the covariants from the bacteriome and mycobiome, species richness, Shannon diversity, community dispersion and FSSC infection status. The response variable was hatchability, unhatched versus hatched eggs, where the unhatched group included failed E.D. and failed L.D. eggs. We used the *ggpredict* function from the R package“ggeffects”^93^ and the *plot* function to visualise the different regression plots. We presented separate plots for each diversity measure modelled with the response variable. Jitter was applied to the data points to show sample size.

#### Are specific bacterial and fungal genera and their MetaCyc metabolic pathways linked with fusariosis infection resistance and hatching success?

To estimate the changes in the relative abundance of specific ASVs between fusariosis infection status and egg development, we first performed ANCOM^23^ analyses across the three egg developmental stages (i.e. failed E.D. eggs as predictors of failed L.D. eggs; failed E.D. eggs as predictors of hatched eggs, and failed L.D. eggs as predictors of hatched eggs), since we did not find significant differences between unhatched failed E.D. and failed L.D. eggs (see Supplementary Fig. 5-7), we analysed this two groups together as“unhatched eggs”. Therefore, our analyses were done on unhatched eggs as predictors of hatched eggs. Second, we analysed fusariosis infection status at each stage of development (i.e. uninfected failed E.D. eggs as predictors of FSSC infected failed E.D. eggs; uninfected failed L.D. eggs as predictors of FSSC infected failed L.D. eggs, and uninfected hatched eggs as predictors of FSSC infected hatched eggs). However, since we did not observe significant differences in all the compared groups (see Supplementary Fig. 6,7), we agglomerated the uninfected samples from the three development stages, and the same was done for the infected samples. For the main results, we analysed uninfected eggs as predictors of FSSC-infected eggs. To visualise the outcomes of ANCOM, we plotted volcano plots of the ANCOM *W* score with respect to the estimates of a GLS controlling for the effect of FSSC infection status (i.e. uninfected and infected) and hatchability (i.e. unhatched and hatched eggs). For FSSC infection status and hatchability, we considered the relative abundance of a specific genera to be differentially abundant according to the predictor only when the *W* was above a threshold of 0.7, a *P* value of < 0.05 from the linear models and when the 95% confidence interval of the GLS estimates did not overlap 0. For the functional predicted pathways we used the PICRUSt2 pipeline^24^ to obtain the predicted MetaCyc metabolic pathways for the bacteriome and for the mycobiome we used the PICRUSt2 pipeline through FROGSFUNC^90^ due to its versatility to perform this analysis. The min-alignment used was 0.8 for the bacteriome and 0.6 for mycobiome The abundance of the predicted MetaCyc pathways was analysed using ANCOM and following the same criteria as described above for taxonomic analyses.

#### Is the complexity of bacterial and fungal interkingdom networks attributed to hatchability?

Following the same approach as the ANCOM analysis, we assessed fusariosis infection status and hatchability (i.e. unhatched and hatched eggs) to identify clusters of co-occurrence interkingdom genera. We examined associations between the 23 most common genera from the bacteriome and the 20 most common genera from the mycobiome using the package NetCoMi^27^. We manually added three taxa for the bacteriome based on the relative abundance results of ANCOM, so our results can be comparable across analysis. We analysed associations between 23 most common bacteria and 20 most common fungi, and only associations with Spearman’s correlation above 0.3 were retained. Co-occurring clusters were identified using the *NetCoMi::netAnalyze* function using the fast and greedy method, and hub pars were calculated based on degree and eigenvector. Clusters show colours, and single clusters or taxa were removed from the network.

### Reporting summary

Further information on research design is available in the Nature Portfolio Reporting Summary linked to this article.

## Supplementary information

Transparent Peer Review file

Supplementary Information

Reporting summary

## Data Availability

All sequences used in this study are stored and available to download under NCBI BioProject PRJNA1199215. The metadata and the code script used for this study are available in Github (https://github.com/DieSofia/Turtle-egg-microbiome-modulates-fusariosis-fungal-infection-and-hatching-success)
