# Supplementary material for: Egg microbiome of the yellow-spotted Amazon river turtle (Podocnemis unifilis) modulates fusariosis fungal infection and hatching success
Source: Commun Biol. 2026 Jun 4;9:830. doi: 10.1038/s42003-026-10404-8 (PMC13275911; doi:10.1038/s42003-026-10404-8)
Supplement: Supplementary file 2 — Supplementary Information [file 42003_2026_10404_MOESM2_ESM.pdf]

Supplementary Information

Egg microbiome of the yellow-spotted Amazon river turtle (*Podocnemis unifilis*) modulates fusariosis fungal infection and hatching success

Ana Sofia Carranco[1,2*], David Romo[2], Maria de Lourdes Torres[3], Kerstin Wilhelm[1], Mark A. F. Gillingham[4#], Simone Sommer[1#*]

[1]Institute of Evolutionary Ecology and Conservation Genomics, University of Ulm, Ulm, Germany.

[2]Tiputini Biodiversity Station, Universidad San Francisco de Quito, Cumbaya–Quito, Diego de Robles y Via Interoceanica s/n, Quito, 170901, Ecuador.

[3]Laboratorio de Biotecnología Vegetal, Universidad San Francisco de Quito USFQ, Diego de Robles y Via Interoceanica s/n, Quito, 170901, Ecuador.

[4]Department of Ornithology, Max Planck Institute for Biological Intelligence, Seewiesen, Germany

#Shared last author

*Ana Sofia Carranco, Simone Sommer
Email: anasoficarranco@gmail.com, Simone.Sommer@uni-ulm.de

This PDF file includes:

Supplementary Note 1
Supplementary Figures 1 to 7
Supplementary Tables 1 to 11

Supplementary Note 1

Abstract in Spanish

La fusariosis en huevos de tortuga, causada por hongos patógenos del complejo de especies *Fusarium solani (*FSSC), constituye una amenaza global para las poblaciones de tortugas debido a la mortalidad embrionaria y el fallo de la eclosión. En vertebrados ovíparos, se ha sugerido que los microbiomas asociados al huésped, incluidos el bacterioma y el micobioma, desempeñan un papel fundamental en el desarrollo de enfermedades; sin embargo, este aspecto ha sido poco explorado. El presente estudio caracteriza el bacterioma y el micobioma del interior de huevos no infectados e infectados por FSSC, recolectados en tres etapas de desarrollo de la tortuga charapa (*Podocnemis unifilis*), una especie vulnerable. Se observó que una menor homogeneidad de especies del micobioma se asocia con una mayor probabilidad e intensidad de infección por FSSC. Independientemente del estado de infección, una mayor diversidad microbiana se relacionó positivamente con el éxito de eclosión, y los huevos que eclosionaron exitosamente presentaron interacciones más complejas entre comunidades bacterianas y fúngicas. Asimismo, se identificaron géneros bacterianos y fúngicos cuya abundancia relativa se asoció negativamente con la infección por FSSC. Estos hallazgos respaldan la hipótesis de que el microbioma del huevo puede influir tanto en la supresión de la infección como en el éxito de eclosión, lo que sugiere el potencial de estrategias de conservación basadas en el microbioma para poblaciones de tortugas amenazadas por patógenos fúngicos.

Supplementary Figures

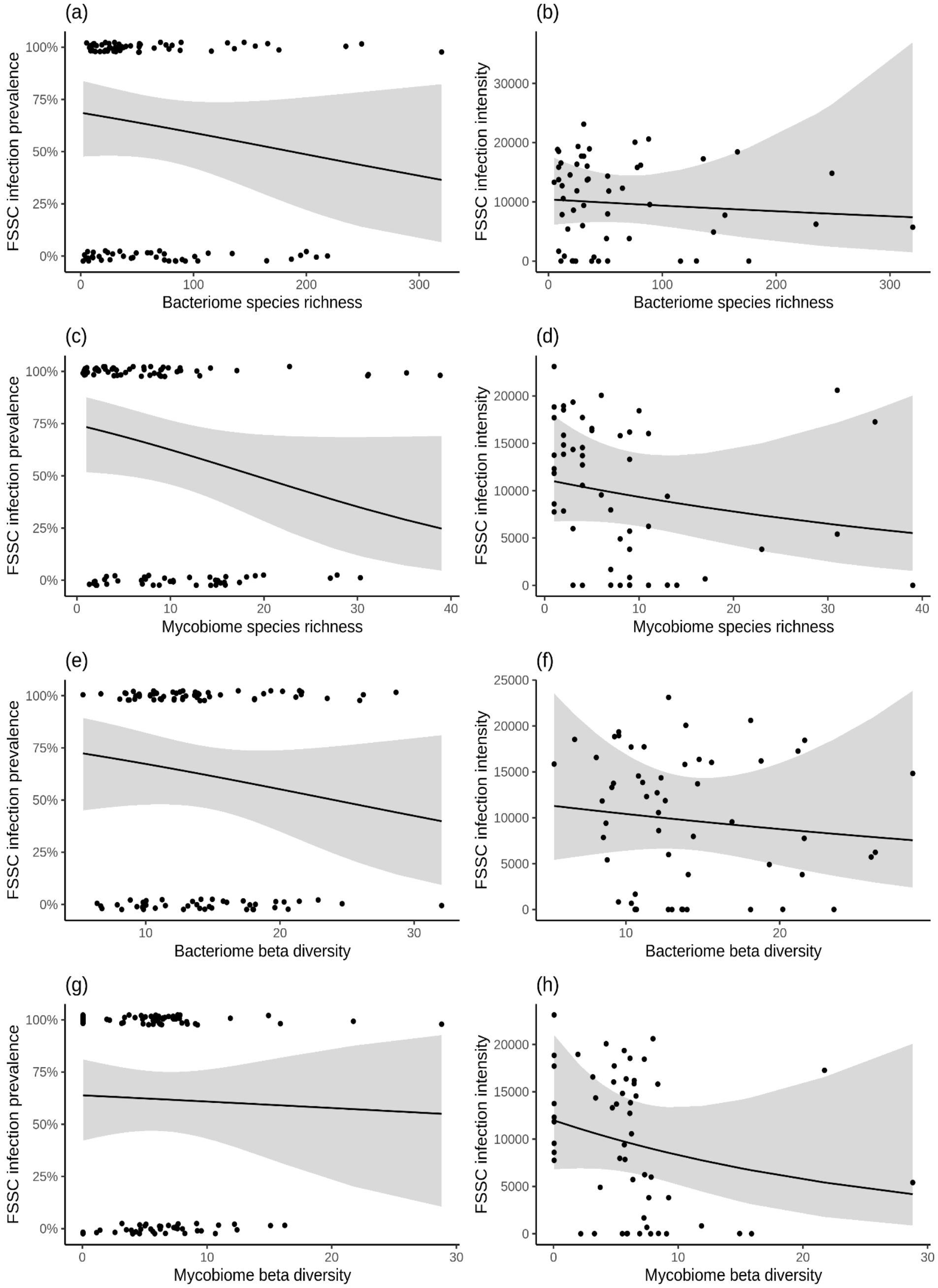

Supplementary Figure 1. Association of bacterial and fungal diversity in turtle eggs with fusariosis infection. Shown are FSSC infection prevalence (in %) and FSSC infection intensity (zero-truncated) according to (a, b) bacteriome species richness, (c, d) mycobiome species richness, (e, f) bacteriome beta diversity, and (g, h) mycobiome beta diversity. The probability of infection is derived from a binomial matrix in which individuals with FSSC ASVs were assigned the number 1, and individuals without FSSC ASVs were assigned the number 0. The intensity of infection derives from the ASV count of the two main species responsible for fusariosis infection (*F. solani* and *F. keratoplasticum*) in each sample of the inner eggshells taken for this study. The fitted line and 95% confidence intervals (shaded area) were modelled using a GLMM with nest ID as a random factor. For the binomial data jitter was applied to the data points to show sample size.

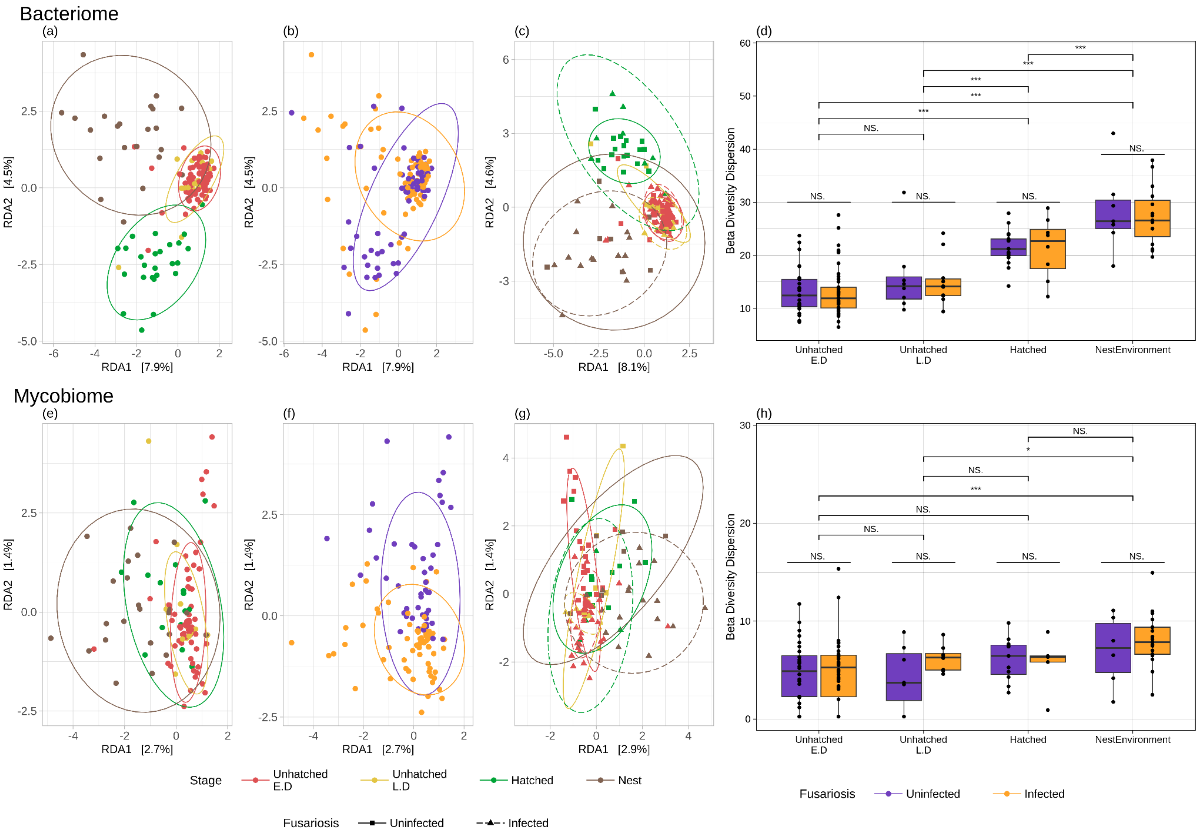

Supplementary Figure 2. Bacterial and fungal composition of inner egg shells in relation to the egg developmental stage and Fusarium infection, including the nesting environment. Redundant analysis (RDA) ordination plots with Euclidean distances of the beta diversity of bacteriomes (upper panels) and mycobiomes (lower panels) according to (a,e) egg developmental stage (failed E.D. (red), failed L.D. (yellow), hatched egg (green)) and nest environmental (brown) samples, (b,f) FSSC infection status (uninfected (purple), infected (orange)), (c,g) the interaction between stage*FSSC, as well as (d,h) box plots of community dispersion using Turkey's multi comparison test for the GLS models. Groups with significant differences are shown with an (*) according to the p-value ("***" 0.001; "**" 0.01; "*" 0.05; "." 0.1), and groups with no significant differences are indicated by the acronym N.S. (not significant).

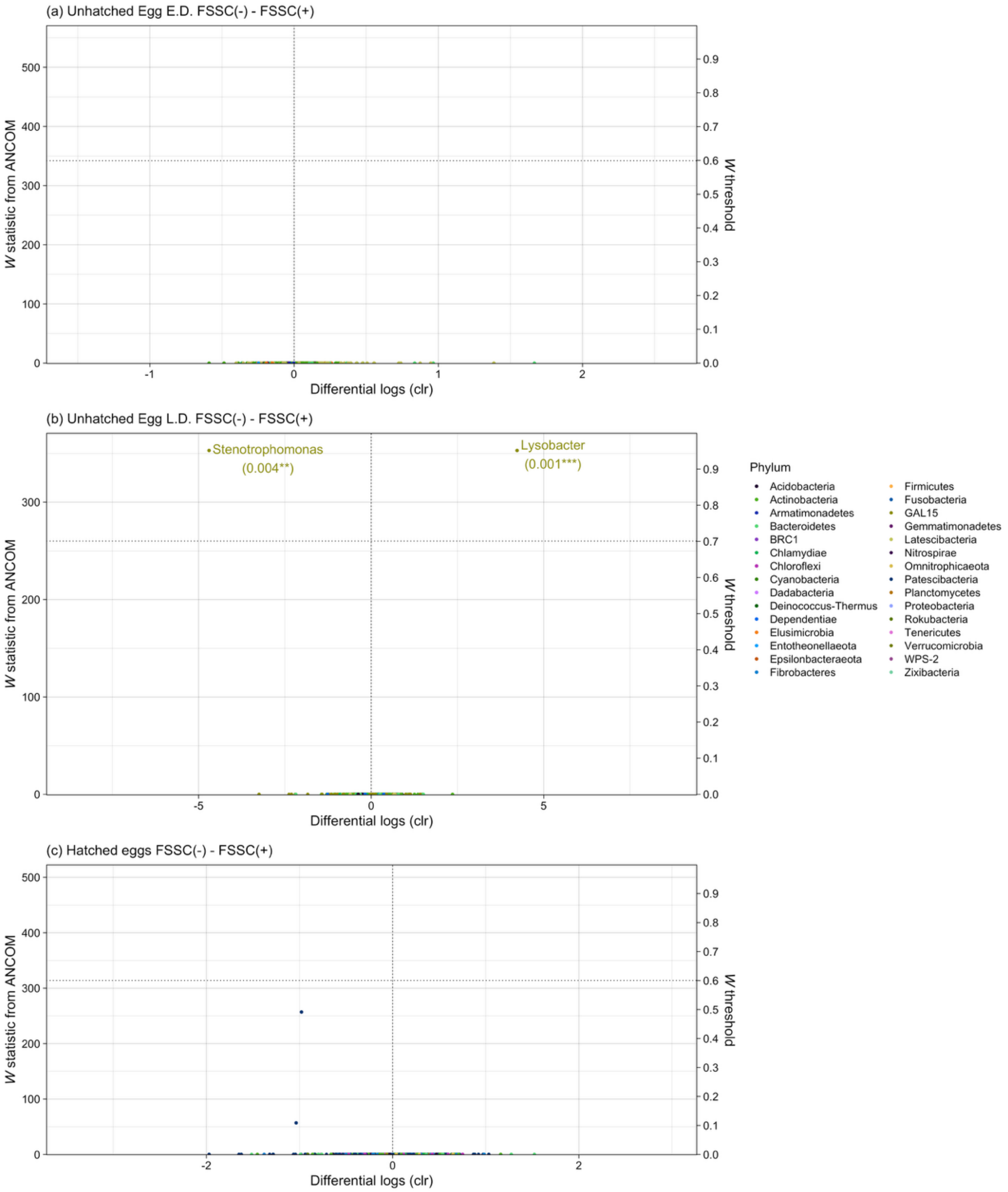

Supplementary Figure 3. Abundance of specific bacterial taxa according to fusariosis infection status in each stage of embryonic development. Volcano plots of ANCOM analyses highlighting bacterial genera differing in abundance between (a–c) FSSC–uninfected and infected eggs. Negative values on the x-axis indicate that a genus is more prevalent in uninfected eggs. The values on the X–axis show the differential log estimates of centred logged ratios (CLR) for a given genus between the different groups. The Y–axis, the *W* statistic, indicates the number of instances where the null hypothesis was rejected for a specific genus. The dots represent individual genera and are coloured according to the phylum level, with genus-level labelling applied when the ANCOM *W* threshold was above 0.7.

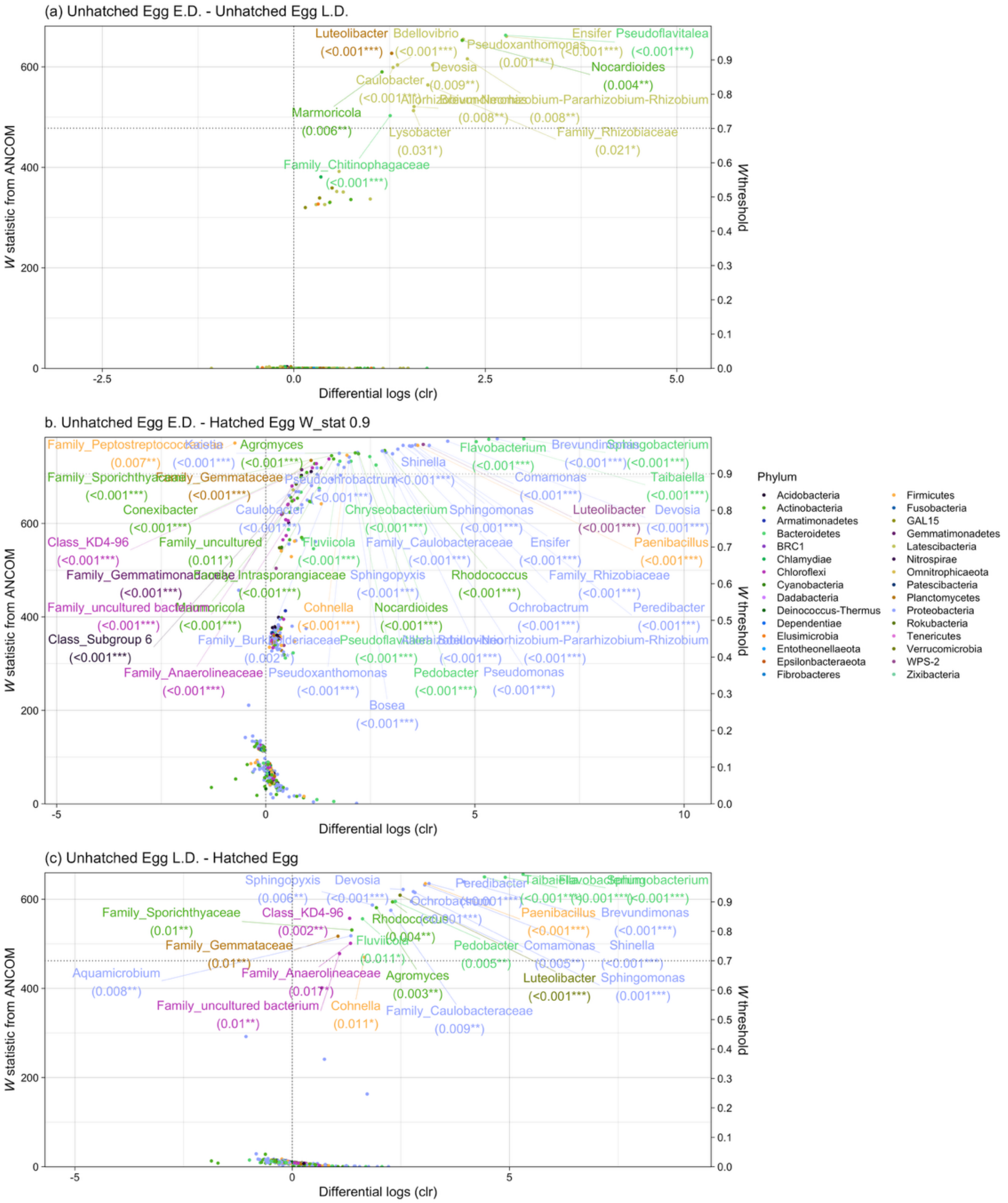

Supplementary Figure 4. Abundance of specific bacterial taxa according to embryonic development. Volcano plots of ANCOM analyses highlighting bacterial genera differing in abundance between failed E.D. eggs and (a) failed L.D. eggs, (b) hatched eggs and (c) between failed L.D. eggs and hatched eggs. Negative values on the x–axis indicate that a genus was more prevalent in failed E.D. and L.D. eggs. The values on the X–axis show the differential log estimates of centred logged ratios (CLR) for a given genus between the different groups. The Y–axis, the *W* statistic, indicates the number of instances where the null hypothesis was rejected for a specific genus. The dots represent individual genera and are coloured according to the phylum level, with genus-level labelling applied when the ANCOM W threshold was above 0.7, except in (b), where the genera were labelled when the ANCOM *W* threshold was above 0.9.

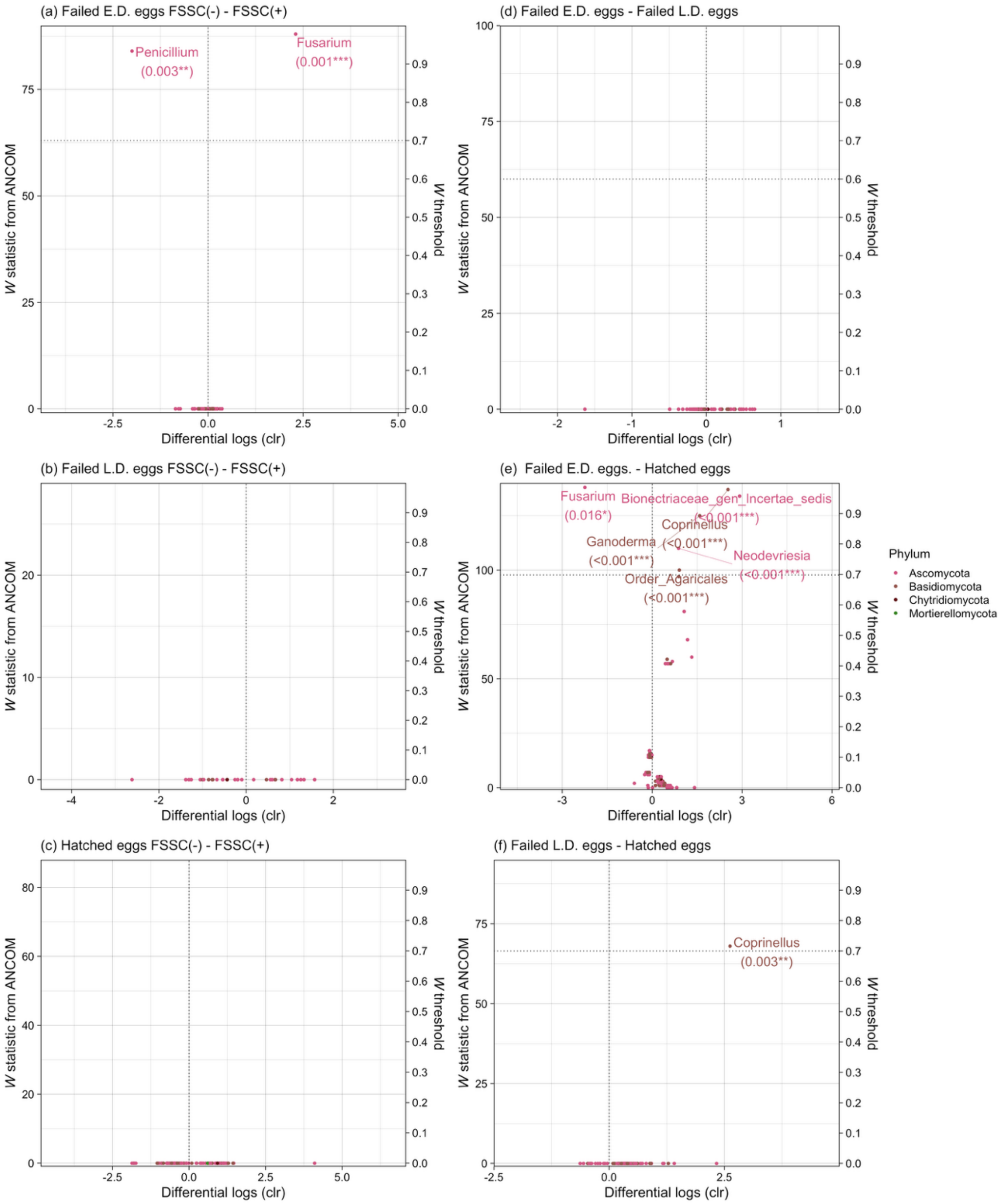

Supplementary Figure 5. Abundance of specific fungal taxa according to infection status and hatching success. Volcano plots of ANCOM analyses highlighting bacterial and fungal genera differing in abundance between (a-c) FSSC–uninfected and infected eggs and (d-f) failed E.D., L.D., and hatched eggs. Negative values on the x-axis indicate that the genus is more prevalent in uninfected and unhatched eggs. The values on the X–axis show the differential log estimates of centred logged ratios (CLR) for a given genus between the different groups. The Y–axis, the *W* statistic, indicates the number of instances where the null hypothesis was rejected for a specific genus. The dots represent individual genera and are coloured according to the phylum level, with genus-level labelling applied when the ANCOM *W* threshold was above 0.7.

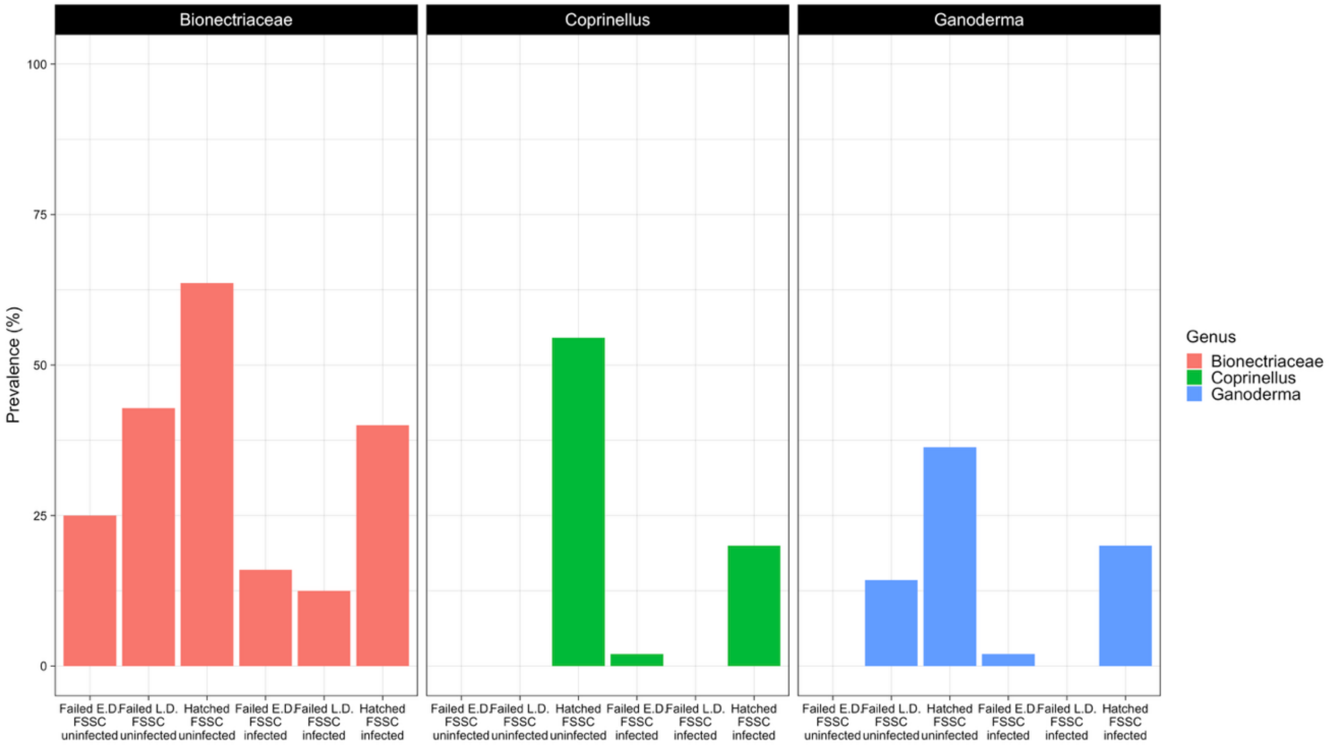

Supplementary Figure 6. Prevalence of the three most differentially abundant fungal genera in hatched eggs according to ANCOM, categorized by developmental stage and FSSC infection status.

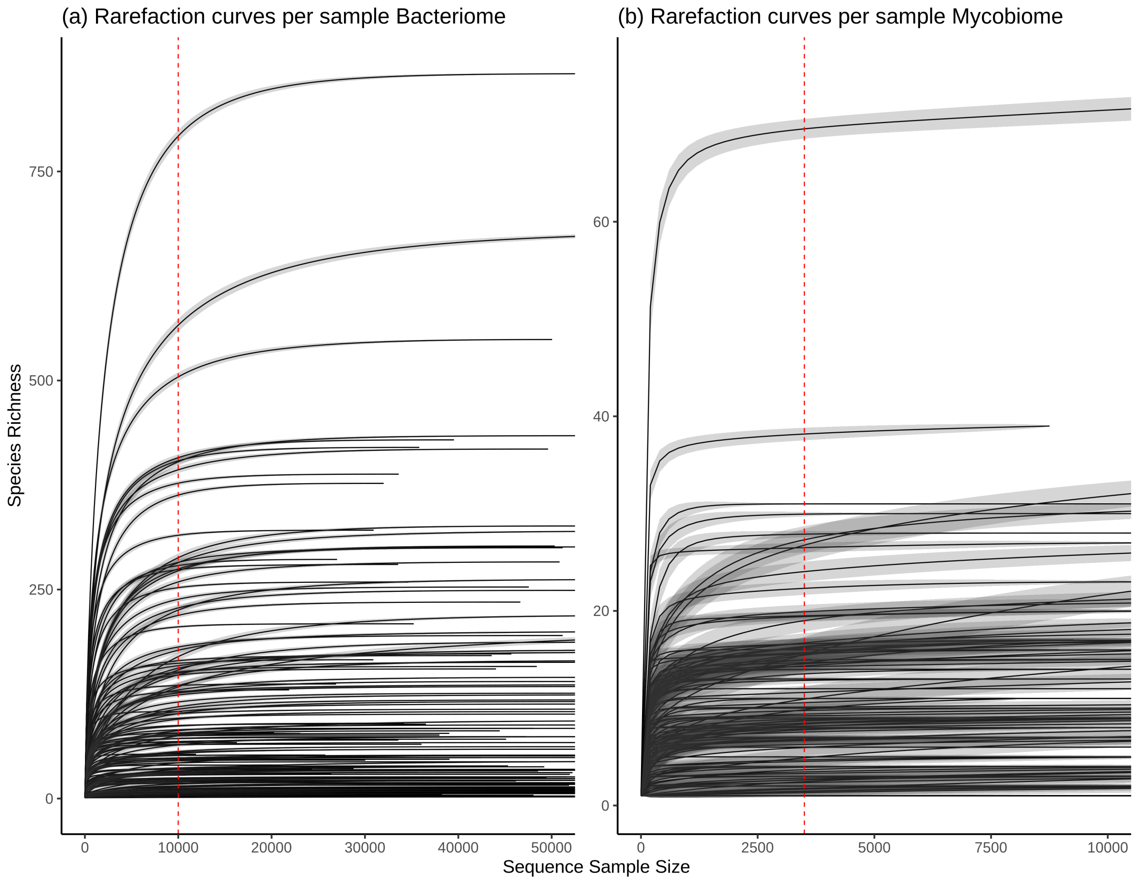

Supplementary Figure 7. Rarefaction curves for the (a) bacteriome and (b) mycobiome showing the diversity richness across samples (samples include inner-eggshells at early and late development and hatched eggs, as well as nest environment samples). The x axis represents the number of sequences sampled while the y axis represents the measure species richness. Dotted red lines indicate where the species richness plateau for each marker.

Supplementary Tables

Supplementary Table 1. Summary of eggs sampled and screened for Fusarium (FSSC) infection, the inner bacteriome and mycobiome. Fusariosis infection status was determined by the presence of FSSC pathogens using metabarcoding of the TEF 1–alpha marker, while the 16S rRNA and ITS markers were employed to characterise the bacteriome and mycobiome, respectively. The mean sequencing coverage and sample sizes (n) are indicated. The development status of the eggs was classified into three categories: (1) dead at an early developmental stage (less than 30 days of incubation, labelled as failed early development (E.D.)), (2) failed to hatch within 100–120 days of incubation with an embryo developed to a late developmental stage (failed late development (L.D.)), and (3) successfully hatched (hatched eggs). The number of nests is indicated in brackets. (/) negative FSSC-PCR test.

Fusariosis infection status	Egg development status	Number of eggs (# of nests)	FSSC sequencing coverage	Bacteriome sequencing coverage	Mycobiome sequencing coverage
uninfected	Failed E.D.	33 (4)	/	51 842 (n = 33)	19 558 (*n* = 28)
	Failed L.D.	8 (1)	/	92 178 (*n* = 8)	17 416 (*n* = 7)
	Hatched	17 (3)	/	61 990 (n = 17)	14 163 (*n* = 11)
infected	Failed E.D.	45 (15)	10 166 (*n* = 45)	46 418 (*n* = 45)	20 386 (*n* = 50)
	Failed L.D.	10 (8)	7126 (*n* = 10)	53 319 (*n* = 10)	17 962 (*n* = 8)
	Hatched	8 (6)	5315 (*n* = 8)	57 944 (*n* = 8)	16 984 (*n* = 5)

Supplementary Table 2. Summary of GLMMs (binomial family and NestID as random factor) testing the effect of fusariosis infection status (uninfected and infected eggs) on bacteriome and mycobiome species richness and beta diversity. Each diversity metric was entered into a separate model. All variables were scaled, and the coefficient (β) and its associated 95% confidence intervals (95%CI) are reported along with the chi-square (χ^2) and p-value statistics.

	β	95%CI	df	χ^2	p-value
Bacteriome species richness	-0.272	[-0.796, 0.253]	1	1.098	0.295
Bacteriome beta diversity	-0.273	[-0.811, 0.266]	1	1.059	0.304
Mycobiome species richness	-0.460	[-0.992, 0.071]	1	3.273	0.070
Mycobiome beta diversity	-0.059	[-0.523, 0.405]	1	0.062	0.803

Supplementary Table 3. Summary of GLMMs (truncated negative binomial family and NestID as a random factor) testing the effect of fusariosis infection intensity (measured as zero-truncated ASV counts of *F. solani* and *F. keratoplasticum* strains) on bacteriome and mycobiome species richness and beta diversity. Each diversity metric was entered into a separate model. All variables were scaled, and the coefficient (β) and its associated 95% confidence intervals (95% CI) were reported along with the chi-square (χ^2) and p-value statistics.

	β	95%CI	df	χ^2	p-value
Bacteriome species richness	-0.071	[-0.470, 0.329]	1	0.115	0.734
Bacteriome beta diversity	-0.090	[-0.470, 0.290]	1	0.208	0.648
Mycobiome species richness	-0.156	[-0.498, 0.187]	1	0.71	0.399
Mycobiome beta diversity	-0.188	[-0.531, 0.155]	1	0.988	0.320

Supplementary Table 4. Model comparison of GLMs (with negative binomial distribution) comparing bacteriome and mycobiome alpha diversity metrics to egg development status (stage = failed E.D, failed L.D, and hatched eggs), *with and without* taking fusariosis infection status (FSSC = uninfected and infected eggs) into account. Species richness of a) bacteriomes and b) mycobiomes, as well as Shannon diversity of c) bacteriomes and d) mycobiomes. See Table S1 for definitions and details. AIC, residuals of degrees of freedom, log-likelihood and deviance are reported along with the model comparison's chi-square ($\chi 2$) or likelihood ratio (LRT) and p-value statistics.

a)	AIC	Resid. df	logLik	df	LRT	p-value
Bacteriome species richness ~ Stage	1235.649	118	-1227.304			
Bacteriome species richness ~ Stage+FSSC	1237.321	117	-1226.799	1	0.505	0.478

b)	AIC	Resid. df	logLik	df	LRT	p-value
Mycobiome species richness ~ Stage	717.287	106	-708.730			
Mycobiome species richness ~ Stage+FSSC	719.115	105	-708.704	1	0.026	0.871

c)	AIC	Resid. df	deviance	df	F-value	p-value
Bacteriome Shannon diversity ~ Stage+FSSC	412.578	117				
Bacteriome Shannon diversity ~ Stage	411.565	118	-0.815	-1	2.568	0.112

d)	AIC	LogLik	deviance	df	χ^2	p-value
Mycobiome Shannon diversity ~ Stage	273.80	-131.90	263.80			
Mycobiome Shannon diversity ~ Stage+FSSC	275.52	-131.76	263.52	1	0.279	0.600

Supplementary Table 5. Model comparison of GLMs (with negative binomial distribution) comparing bacteriome and mycobiome alpha diversity metrics to egg development status (stage = failed E.D, failed L.D, and hatched eggs), *with and without taking the interaction* term with fusariosis infection status (FSSC = uninfected and infected eggs) into account. Species richness of a) bacteriomes, and b) mycobiomes, as well as Shannon diversity of c) bacteriomes and d) mycobiomes. See Table S1 for definitions and details. AIC, residuals of degrees of freedom, log-likelihood and deviance are reported along with the model comparison's chi-square (χ2) or likelihood ratio (LRT) and p-value statistics.

a)	AIC	Resid. df	logLik	df	LRT	p-value
Bacteriome species richness ~ Stage+FSSC	1237.321	117	-1226.799			
Bacteriome species richness ~ Stage*FSSC	1241.324	115	-1226.332	2	0.467	0.792

b)	AIC	Resid. df	logLik	df	LRT	p-value
Mycobiome species richness ~ Stage+FSSC	719.287	105	-708.704			
Mycobiome species richness ~ Stage*FSSC	723.412	103	-708.303	2	0.401	0.818

c)	AIC	Resid. df	deviance	df	F-value	p-value
Bacteriome Shannon diversity ~ Stage*FSSC	416.892	115				
Bacteriome Shannon diversity ~ Stage+FSSC	412.578	117	-0.109	-2	0.167	0.847

d)	AIC	LogLik	deviance	df	χ^2	p-value
Mycobiome Shannon diversity ~ Stage+FSSC	275.52	-131.76	263.52			
Mycobiome Shannon diversity ~ Stage*FSSC	279.37	-131.69	263.37	2	0.144	0.931

Supplementary Table 6. Results of ANOVA RDA analysis comparing variation in bacterial community structure to a) egg development status (stage = failed E.D, failed L.D, and hatched eggs), b) fusariosis infection status (FSSC = uninfected and infected eggs), and c) the interaction between stage and fusariosis infection status. See Table S1 for definitions and details. P-values <0.05 are represented in bold.

a)	Df	Variance	F	Pr(>F)
Model	2	24.332	6.073	**1e-05 *****
Residual	118	236.390		

b)	Df	Variance	F	Pr(>F)
Model	1	2.916	1.3458	0.08117 .
Residual	119	257.806		

c)	Df	Variance	F	Pr(>F)
Model	5	31.543	3.1656	**1e-05 *****
Residual	115	229.179		

Supplementary Table 7. Pairwise comparisons of RDA using constrained ordination analysis with Euclidean distances of the bacteriome using the variables a) egg development status (stage = failed E.D, failed L.D, and hatched eggs), b) fusariosis infection status (FSSC uninfected and infected eggs) and c) the interaction between stage and fusariosis infection status.

a)	Group pairs	Df	Sum of Sqs	F	Pr(>F)
	FailedED vs. FailedLD	1	1.304	0.976	0.410
	FailedED vs. HatchedEgg	1	23.971	16.399	**0.001****
	FailedLD vs. HatchedEgg	1	27.288	5.885	**0.001****

b)	Group pairs	Df	Sum of Sqs	F	Pr(>F)
	Uninfected vs. FSSC infected	1	2.063	1.479	**0.086 .**

c)	Group pairs	Df	Sum of Sqs	F	Pr(>F)
	Failed E.D.-Uninfected vs. Failed E.D.-Infected	1	1.006	0.631	0.900
	Failed E.D.-Uninfected vs. Failed L-D.-Uninfected	1	6.805	2.120	**0.021***
	Failed E.D.-Uninfected vs. Failed L-D.-Infected	1	2.347	0.759	0.755
	Failed E.D.-Uninfected vs. Hatched-Uninfected	1	31.145	9.725	**0.001*****
	Failed E.D.-Uninfected vs. Hatched-Infected	1	24.543	6.731	**0.001*****
	Failed E.D.-Infected vs. Failed L-D.-Uninfected	1	4.456	1.746	0.043
	Failed E.D.-Infected vs. Failed L-D.-Infected	1	2.296	0.981	0.438
	Failed E.D.-Infected vs. Hatched-Uninfected	1	26.191	10.550	**0.001*****
	Failed E.D.-Infected vs. Hatched-Infected	1	19.261	6.863	**0.001*****

Failed L-D.-Uninfected vs. Failed L-D.-Infected	1	15.441	1.691	**0.016***
Failed L-D.-Uninfected vs. Hatched-Uninfected	1	17.690	2.160	**0.007****
Failed L-D.-Uninfected vs. Hatched-Infected	1	26.219	2.012	**0.027****
Failed L-D.-Infected vs. Hatched-Uninfected	1	39.346	5.380	**0.001*****
Failed L-D.-Infected vs. Hatched-Infected	1	48.329	4.324	**0.001*****
Hatched-Uninfected vs. Hatched-Infected	1	7.778	0.867	0.599

Supplementary Table 8. Results of ANOVA RDA analysis comparing variation in fungal community structure to a) egg developmental status (stage = failed E.D, failed L.D, and hatched eggs), b) fusariosis infection status (FSSC = uninfected and infected eggs), and c) the interaction between stage*FSSC. See Table S1 for definitions and details. P-values <0.05 are represented in bold.

a)	Df	Variance	F	Pr(>F)
Model	2	0.995	0.918	0.5697
Residual	106	57.432		

b)	Df	Variance	F	Pr(>F)
Model	1	0.833	1.5481	**0.001****
Residual	107	57.593		

c)	Df	Variance	F	Pr(>F)
Model	5	2.548	0.9392	0.5569
Residual	103	55.879		

Supplementary Table 9. Model selection of a) bacteriome and b) mycobiome beta diversity dispersion using GLS (with maximum likelihood) using the variables Stage (three different stages of egg development, failed eggs E.D. and L.D., and hatched eggs) and fusariosis infection status (uninfected and infected eggs).

a)	Intercept	FSSC	Stage	df	logLik	AICc	**Δ**AICc	weight	adjR^2
3	12.973		+	5	-356.781	724.083	0.000	0.752	0.352
4	12.979	+	+	6	-356.780	726.298	2.215	0.248	0.352
1	15.065			3	-382.859	771.924	47.841	0.000	0.002
2	15.827	+		4	-381.975	772.296	48.213	0.000	0.016

b)	Intercept	FSSC	Stage	df	logLik	AICc	**Δ**AICc	weight	adjR^2
1	6.308			4.000	-316.907	642.199	0.000	0.531	0.147
3	5.935		+	6.000	-315.579	643.982	1.783	0.218	0.168
2	6.380	+		5.000	-316.890	644.363	2.164	0.180	0.147
4	5.820	+	+	7.000	-315.552	646.213	4.014	0.071	0.168

Supplementary Table 10. GLMM model comparisons (binomial family) testing the effect of bacterial and fungal diversity on hatchability (i.e. successful hatching) *with and without the interaction* term with FSSC infection status. The microbiome diversity metrics for the bacteriome and mycobiome were species richness, Shannon diversity and beta diversity dispersion. Each diversity metric was entered into a separate model. Models with the FSSC interaction are shown with (*), and models without the FSSC interaction are shown with (-). All variables were scaled, and the AIC, BIC, log-likelihood and deviance were reported along with the model comparison's chi-square ($\chi 2$) and p-value statistics.

	npar	AIC	BIC	logLik	deviance	df	χ^2	p-value
+Bacteriome species richness (-)	4	64.687	74.986	-28.343	56.687			
*Bacteriome species richness (*)	5	63.549	76.423	-26.744	53.549	1	3.138	0.077
+Bacteriome Shannon diversity (-)	4	65.328	75.627	-28.664	57.328			
*Bacteriome Shannon diversity (*)	5	67.081	79.954	-28.540	57.081	1	0.248	0.619
+Bacteriome beta diversity (-)	4	69.638	79.936	-30.819	61.638			
*Bacteriome beta diversity (*)	5	71.332	84.206	-30.666	61.332	1	0.305	0.581
+Mycobiome species richness (-)	4	65.577	75.876	-28.788	57.577			
*Mycobiome species richness (*)	5	67.431	80.305	-28.716	57.431	1	0.145	0.703
+Mycobiome Shannon diversity (-)	4	63.104	73.403	-27.522	55.104			
*Mycobiome Shannon diversity (*)	5	65.093	77.966	-27.546	55.093	1	0.011	0.915
+Mycobiome beta diversity (-)	4	79.275	89.574	-35.637	71.275			
*Mycobiome beta diversity (*)	5	81.123	93.996	-35.561	71.123	1	0.152	0.697

Supplementary Table 11. GLMM model comparisons (binomial family) testing the effect of bacterial and fungal diversity on hatchability (i.e. hatching success) *with and without* the variable FSSC infection status. The microbiome diversity metrics for the bacteriome and mycobiome were species richness, Shannon diversity and beta diversity dispersion. Each diversity metric was entered into a separate model. Models with the variable FSSC are shown with (+), and models without the variable FSSC are shown with (-). All variables were scaled, and the AIC, BIC, log-likelihood and deviance were reported along with the model comparison's chi-square ($\chi 2$) and p-value statistics.

	npar	AIC	BIC	logLik	deviance	df	χ^2	p-value
Bacteriome species richness (-)	3	70.518	78.242	-32.259	64.518			
Bacteriome species richness (+)	4	64.687	74.986	-28.343	56.687	1	7.832	**0.005****
Bacteriome Shannon diversity (-)	3	72.252	79.976	-33.126	66.252			
Bacteriome Shannon diversity (+)	4	65.328	75.627	-28.664	57.328	1	8.924	**0.003****
Bacteriome beta diversity (-)	3	76.146	83.870	-35.073	70.146			
Bacteriome beta diversity (+)	4	69.638	79.936	-30.819	61.638	1	8.51	**0.004****
Mycobiome species richness (-)	3	71.729	79.453	-32.864	65.729			
Mycobiome species richness (+)	4	65.577	75.876	-28.788	57.577	1	8.152	**0.004****
Mycobiome Shannon diversity (-)	3	65.362	73.086	-29.681	59.362			
Mycobiome Shannon diversity (+)	4	63.104	73.403	-27.552	55.104	1	4.258	**0.040***
Mycobiome beta diversity (-)	3	86.879	94.603	-40.440	80.879			
Mycobiome beta diversity (+)	4	79.275	89.574	-35.637	71.275	1	9.605	**0.002****
