# Supplementary material for: Egg microbiome of the yellow-spotted Amazon river turtle (Podocnemis unifilis) modulates fusariosis fungal infection and hatching success
Source: Commun Biol. 2026 Jun 4;9:830. doi: 10.1038/s42003-026-10404-8 (PMC13275911; doi:10.1038/s42003-026-10404-8)
Supplement: Supplementary file 3 — Reporting summary [file 42003_2026_10404_MOESM3_ESM.pdf]

nature portfolio

Corresponding author(s): Ana Sofia Carranco

Last updated by author(s): May 15, 2026

Reporting Summary

Nature Portfolio wishes to improve the reproducibility of the work that we publish. This form provides structure for consistency and transparency in reporting. For further information on Nature Portfolio policies, see our Editorial Policies and the Editorial Policy Checklist.

Statistics

For all statistical analyses, confirm that the following items are present in the figure legend, table legend, main text, or Methods section.

n/a	Confirmed	
☐	☒	The exact sample size (*n*) for each experimental group/condition, given as a discrete number and unit of measurement
☐	☒	A statement on whether measurements were taken from distinct samples or whether the same sample was measured repeatedly
☐	☒	The statistical test(s) used AND whether they are one- or two-sided<br>*Only common tests should be described solely by name; describe more complex techniques in the Methods section.*
☐	☒	A description of all covariates tested
☐	☒	A description of any assumptions or corrections, such as tests of normality and adjustment for multiple comparisons
☐	☒	A full description of the statistical parameters including central tendency (e.g. means) or other basic estimates (e.g. regression coefficient) AND variation (e.g. standard deviation) or associated estimates of uncertainty (e.g. confidence intervals)
☐	☒	For null hypothesis testing, the test statistic (e.g. *F*, *t*, *r*) with confidence intervals, effect sizes, degrees of freedom and *P* value noted<br>*Give P values as exact values whenever suitable.*
☒	☐	For Bayesian analysis, information on the choice of priors and Markov chain Monte Carlo settings
☒	☐	For hierarchical and complex designs, identification of the appropriate level for tests and full reporting of outcomes
☐	☒	Estimates of effect sizes (e.g. Cohen's *d*, Pearson's *r*), indicating how they were calculated

Our web collection on statistics for biologists contains articles on many of the points above.

Software and code

Policy information about availability of computer code

Data collection	Samples were collected at the Tiputini Biodiversity Station and transported to Germany at the Ulm University where they were processed and sequenced. A database was created with all the sample information.
Data analysis	Bioinformatic and statistical analyses were performed using the software R (v4.2.3 R Core Team 2022). The R code including all packages used for the analyses of the present study are available on GitHub.

For manuscripts utilizing custom algorithms or software that are central to the research but not yet described in published literature, software must be made available to editors and reviewers. We strongly encourage code deposition in a community repository (e.g. GitHub). See the Nature Portfolio guidelines for submitting code & software for further information.

Data

Policy information about availability of data

All manuscripts must include a data availability statement. This statement should provide the following information, where applicable:

- Accession codes, unique identifiers, or web links for publicly available datasets
- A description of any restrictions on data availability
- For clinical datasets or third party data, please ensure that the statement adheres to our policy

All the RAW seqeunces are available in NCBI under the accession number: BioProject PRJNA1199215 and will be public after acceptance.

The metadatas and scripts used in the current study are available on GitHub https://github.com/DieSofia/Turtle-egg-microbiome-modulates-fusariosis-fungal-infection-and-hatching-success

Research involving human participants, their data, or biological material

Policy information about studies with human participants or human data. See also policy information about sex, gender (identity/presentation), and sexual orientation and race, ethnicity and racism.

Reporting on sex and gender	Not applicable
Reporting on race, ethnicity, or other socially relevant groupings	Not applicable
Population characteristics	Not applicable
Recruitment	Not applicable
Ethics oversight	Not applicable

Note that full information on the approval of the study protocol must also be provided in the manuscript.

Field-specific reporting

Please select the one below that is the best fit for your research. If you are not sure, read the appropriate sections before making your selection.

☐ Life sciences ☐ Behavioural & social sciences ☒ Ecological, evolutionary & environmental sciences

For a reference copy of the document with all sections, see nature.com/documents/nr-reporting-summary-flat.pdf

Ecological, evolutionary & environmental sciences study design

All studies must disclose on these points even when the disclosure is negative.

Study description	In this study, we used the yellow-spotted Amazon river turtle to investigate the potential role of the bacterial and fungal communities of the inner eggshell in defending against fungal pathogens and enhancing hatching success. To do this, we collected internal eggshell swab samples from 121 eggs, categorised into three different developmental stages. Fusariosis infection was initially determined visually; eggs displaying signs of fungal infection were categorised as infected, while those without signs were marked as uninfected. During laboratory analysis, egg swab samples were tested for Fusariosis infection using specific Fusarium spp. primers for the TEF-alpha region and subsequent sequencing. We amplified and sequenced the 16S V4 and ITS regions from all samples using an Illumina MiSeq platform to analyse and quantify the bacterial and fungal communities. With the collected data, we sought to determine whether the inner egg bacterial and fungal diversity predicts the prevalence and intensity of Fusariosis infection. Next, we assessed whether bacterial and fungal diversity and composition differed between eggs that had undergone arrested development at early and late embryonic stages compared to successfully hatched eggs. Furthermore, we tested the effect of Fusariosis infection and its interaction with developmental stage. We also investigated whether bacterial and fungal diversity are predictors of hatching success. Finally, we aimed to detect whether specific bacteria and fungi and their interactions are linked to FSSC suppression/ susceptibility and hatching success.
Research sample	The eggs of the yellow-spotted Amazon river turtle were selected after a 100–120-day incubation period. Sampling was based on the hatching activity of each nest. Successfully hatched eggs were prioritized for sampling, as our goal was to obtain samples from the inner eggshell of freshly hatched eggs to avoid contamination from the surrounding sand environment. We then selected unhatched eggs and carefully examined whether they had fractures or were intact. Only intact eggs were considered for sampling. From the unhatched eggs, we first conducted a visual inspection for signs of fusariosis fungal infection. If the eggs exhibited symptoms of fusariosis infection (green, black, yellow, or pink dots), they were categorized as "visually infected"; if they showed no signs of infection, they were categorized as "uninfected". For collecting samples from unhatched eggs, we carefully opened the egg and swabbed the inner eggshell. Next we checked for any signs of development. If we noticed a small portion of the egg enclosed by the lumen or infected by fungi, we classified this as having failed to hatch at an early developmental stage. If we observed a visibly fully formed embryo in the egg with interrupted development, we determined this as having failed to hatch at a late developmental stage.
Sampling strategy	Eggs were carefully selected for sampling and manipulated using sterile gloves. Eggs were taken from the nest, and any remaining sand from the nesting environment was removed with fingers covered by sterile gloves. Eggshells were opened, and a sterile swab was inserted to swab the internal eggshell. Samples were collected by continuous swabbing for approximately five to ten seconds.
Data collection	Data collection was performed by A.S.C during field work. During laboratory work, A.S.C collected the data with assistance of K.W. A database of the sample collection was done by A.S.C.
Timing and spatial scale	The data was collected from artificial nests of the yellow-spotted Amazon river turtle at the Tiputini Biodiversity Station from the laying season 2018-2019. After the incubation period, sample collection started in March 2019 and finished in May 2019.
Data exclusions	After preprocessing and filtering out bacterial and fungal contaminants from control samples (field blanks, extraction blanks, and PCR

Data exclusions	blanks), samples with fewer than 10,000 ASV reads for the bacterial microbiome, and 3,000 reads for the fungal microbiome were excluded from further analyses. For the network analysis, only samples successfully screened for bacterial and fungal communities were included. If samples contained data from the bacterial microbiome but not the fungal microbiome, or vice versa, those samples were not considered for the network analysis.
Reproducibility	During fieldwork, not all the nests successfully produced hatchlings; some nests had more infected eggs than others. Consequently, we collected eggs from all the nests, aiming to account for the number of eggs per nest and treatment when possible (i.e., four eggs per nest).
Randomization	Samples were initially categorized by the nest, which was subsequently used to control the random factor in the models. Fungal infection was assessed visually, while fusariosis infection was ultimately determined through TEF alpha gene PCR and sequencing. Development was not considered in randomization, as developmental stage was identified after opening the egg.
Blinding	All laboratory work was performed blind to the identity of the sample.

Did the study involve field work? ☒ Yes ☐ No

Field work, collection and transport

Field conditions	Fieldwork was conducted in the Amazon region of Ecuador within a primary forest during the rainy season from March to May 2019. Humidity fluctuated between 95% and 100%, while temperatures ranged from 25°C to 39°C. The artificial nests were incubated in plastic boxes filled with sand collected from the natural area. All the nests were situated on a platform at the Tiputini Biodiversity Station in an open space with partial canopy cover near the river basin. The platform was approximately 1.2 m above the ground to protect the nests from rising water levels. It was also covered with a roof made of greenhouse plastic lifted about 1.6 m above the platform to allow airflow, sunlight, and some rain to reach the nests.
Location	The Tiputini Biodiversity Station from Universidad San Francisco de Quito is located in the Yasuni Biosphere Reserve, Orellana Province, 280 km ESE of Quito, Ecuador (0 °38'18"S 76°9'0"w).
Access & import/export	We collaborated with the Tiputini Biodiversity Station's reintroduction programme for the yellow-spotted Amazon river turtle and worked with a parabiologist from the station. Our research fell under the collection permit from M.L.T. at Universidad San Francisco de Quito, permit no. MAE-DNB-CM-2016-0046-M-0003. After collecting and preparing the material for export to Germany, we issued the export permit no. 158-2019-EXP-CM-FAU-DNB/MA. Both permits were granted by the Environmental Ministry of Ecuador.
Disturbance	Samples were taken after the incubation period to minimize any disturbance to the incubating embryos. When turtles were hatching, we carefully checked whether the hatchling had completely left the egg or was still in the hatching process. We aimed to collect samples after the turtle had left the egg. During sampling of the unhatched eggs, we waited two weeks after the normal time of hatching (90 days) to avoid disturbing embryos that had delayed hatching. Eggs that were visibly underdeveloped and infected were immediately removed from the nest and sampled.

Reporting for specific materials, systems and methods

We require information from authors about some types of materials, experimental systems and methods used in many studies. Here, indicate whether each material, system or method listed is relevant to your study. If you are not sure if a list item applies to your research, read the appropriate section before selecting a response.

Materials & experimental systems

n/a	Involved in the study
☒	☐ Antibodies
☒	☐ Eukaryotic cell lines
☒	☐ Palaeontology and archaeology
☐	☒ Animals and other organisms
☒	☐ Clinical data
☒	☐ Dual use research of concern
☒	☐ Plants

Methods

n/a	Involved in the study
☒	☐ ChIP-seq
☒	☐ Flow cytometry
☒	☐ MRI-based neuroimaging

Animals and other research organisms

Policy information about studies involving animals; ARRIVE guidelines recommended for reporting animal research, and Sex and Gender in Research

Laboratory animals	The study did not involve laboratory animals.
Wild animals	Eggs were collected in the wild and then transported to the research station. After the incubation period, samples from hatched eggs

Wild animals	and eggs that failed to hatch (unhatched eggs) were taken. Juvenile turtles were later on released to the Tiputini river at the sites were the nests were originally collected.
Reporting on sex	Not applicable.
Field-collected samples	Sample collection was conducted after the egg incubation period, and the eggs were categorised into three different developmental stages: those that failed to hatch at an early and late stage of development, and those that successfully hatched. The eggs were handled with sterile gloves and carefully removed from the nest environment. Remaining sand within the egg was delicately cleared using gloved hands, and then the eggs were gently opened. Following the opening, a sterile swab was inserted and made contact with the internal eggshell; samples were collected by continuous swabbing for 5 to 10 seconds. The swabs were promptly placed into 1.5 µl Eppendorf tubes containing Nucleic Acid Preservation (NAP) buffer and stored at room temperature during fieldwork. Upon transport to the laboratories of the Institute of Evolutionary Ecology and Conservation Genomics at Ulm University, the samples were stored in a refrigerator at 4 °C before sequencing. DNA extraction was conducted using a NucleoSpin 96 Soil extraction kit (Macherey-Nagel, Germany).
Ethics oversight	The Tiputini Biodiversity Station at Universidad San Francisco de Quito approved this study. The Tiputini Biodiversity Station has been running the reintroduction project since 2007 with permission from the Ethics Committee at Universidad San Francisco de Quito.

Note that full information on the approval of the study protocol must also be provided in the manuscript.

Plants

Seed stocks	Not applicable
Novel plant genotypes	Not applicable
Authentication	Not applicable
