# Supplementary material for: Egg microbiome of the yellow-spotted Amazon river turtle (Podocnemis unifilis) modulates fusariosis fungal infection and hatching success
Source: Commun Biol. 2026 Jun 4;9:830. doi: 10.1038/s42003-026-10404-8 (PMC13275911; doi:10.1038/s42003-026-10404-8)
Supplement: Supplementary file 1 — Transparent Peer Review file [file 42003_2026_10404_MOESM1_ESM.pdf]

nature portfolio

Peer Review File

Egg microbiome of the yellow-spotted Amazon river turtle (Podocnemis unifilis) modulates fusariosis fungal infection and hatching success

Corresponding Author: Ms Ana Carranco

This manuscript has been previously submitted at another journal. This document only contains information relating to versions considered at Communications Biology.

This file contains all reviewer reports in order by version, followed by all author rebuttals in order by version.

Version 0:

Reviewer comments:

Reviewer #1

(Remarks to the Author)
Carranco et al. present “Turtle egg microbiome modulates fusariosis fungal infection and hatching success” for review. This manuscript presents novel hypothesis driven research examining the relationships between the bacteriome, mycobiome, and Fusarium infection status in Podocnemis unifilis eggs over developmental time. The manuscript is generally well written. My only major concern is methodological. Namely how internal egg samples/swabs are taken; if it the same as papers cited, then it seems that the inside of the shell is swabbed after breaking/hatching, which seems to risk sampling external microbes (air, etc.) and not any or only a partial internal microbiome (please clarify if this is not correct). This method and all controls used are therefore important to address as there remains skepticism in the microbiome community of microbes existing/thriving in places like the inside of a reptile egg. Also, if there are truly microbes inside the egg and the egg shell is impermeable to microbes, where are the microbes coming from initially? If from inside the mother, then this seems vertical transmission is likely.

There are also a few minor clarifications, suggestions, and edits that are outlined below:

Figure 1 should be changed/altered as it is partly the same figure from https://onlinelibrary.wiley.com/doi/full/10.1111/mec.16548

Line 28-29: Consider “development” or some derivative in keywords as the developmental component of this work seems important.

Line 69-73: Is there a turtle microbiome paper showing possible vertical transmission that could be cited here? It seems possible that this is happening in your system, although that wasn’t examined. I think this possibility should be highlighted in discussion.

Line 79: Is it possible some of the bacteria or fungi are contaminants from air, the outside of shell, or sampling equipment? How was this controlled for (in methods)?

Line 98: “100-120 days” should have an en dash, make sure to use throughout

Line 105-108: Maybe mention here or in methods that keeping nest as a random effects will help to deal with the issue of pseudoreplication (multiple eggs coming from the same nest). Based on your method of sampling, no individual egg was ever sampled more than once, correct?

Line 123-125: You might try structural equation models (SEMs) to assign directionality to the effect.

Line 188-190: The nest looks different (including significantly different in beta diversity dispersion) in various panels of supp

figure S1. So can you explain more how this figure supports the non-nest findings? Or clarify what you are saying here.

Line 296: I think the discussion is good overall, but I would consider adding one or two sentences about possible vertical transmission in your system, maybe as a point to be followed-up on. Especially in Line 315-318, is it possible there is an external shell microbiome that is vertically transferred and then inherited upon hatching?

Line 340-342: The microbial ecology theory application to Anna Karenina is well-placed and is appreciated.

Line 363-364: This would seem to align well with vertical transmission/holobiont theory.
Line 451: Where do you think Fusarium is originating in turtles? Was it found in your nest samples also (external environment)?

Line 534-535: Were positive PCR controls also included? For example the Zymo microbial community DNA? Or some known amount of microbial DNA?

Line 564: What were the max reads and average reads per sample? Was rarefaction used or not? I would state explicitly one way or the other so it is clear.

In supplemental methods line 115-116, you use the Silva database. Consider using Greengenes2 as this is much improved over Silva version 132. https://www.nature.com/articles/s41587-023-01845-1

Reviewer #2

(Remarks to the Author)
The study investigates whether the inner egg microbiome (bacterial and fungal communities) of the yellow-spotted Amazon river turtle influences susceptibility to Fusarium fungal infection and hatching success by comparing infected vs uninfected eggs across developmental stages.

Good job to authors for a very careful experimental design! I understand the limitation of using only 16S and ITS markers for the functional study. With the design, a metagenomic approach would definitely helps to advances the understanding of this research (Maybe future work? :P)

General comments:
1. Infected samples, no matter ED or LD, were collected after FSSC-infected symptoms had developed, which means authors are comparing community diversity of healthy vs. diseased eggs at post-infection. Does it also means authors are assumming pre-infected eggs also already had lower diversity? Hence, it is unclear whether the observed microbiome differences arise early and influence outcomes, or emerge later as a consequence of embryo death, infection progression, or hatching.

2. On a conservation practice perspective, with your study results, can you suggest what is the next step (application or research) that may helps improving the hatchability of this turtle in the artificial incubation settings?

Introduction:
Line 57: Last sentence. I would suggest to describe specifically about "host-protective traits" (i.e. host at the egg stage) .

Methods:
Line 480: Just curious if the sand "controlled environment" for keeping the eggs is sterilized? If no, how do you think sand from the hatchery environment will affect your result?

Line 551: What was the "significant consequences" of the ITS region length variation here means?

Line 554: cutadapt spelling error

Line 572: "we only took the information of the infected samples with an FSSC ASV count greater than one and added it as a variable..." Can you explain again what this sentence means? I do not quite get it.

For paragraph Taxonomic assignment of Fusarium (FSSC) pathogens in Line 566: Maybe it's also best to mention that ASV is look at the FSSC level instead of each species (Ff, Fk, Fs) because their sequences are too similar etc., as an additional information for readers not familiar with this taxa

I think Line 175, sentences from the supplementary file: "Only sequences assigned to Fusarium or the teleomorph name (Gibberella and Nectria) longer than 300 were selected for further analysis" is important information to be included to the method in the main article. Consider including it?

Supplementary file:
Almost all supporting figures are too difficult to read.

Version 1:

Reviewer comments:

Reviewer #1

(Remarks to the Author)
The revised manuscript appears to address my previous comments, and provides context/limitations where appropriate. No further comments at this time.

Reviewer #2

(Remarks to the Author)
Dear authors,

Thank you for the revision. All my previous concerns and questions have been adequately addressed, and I have no further questions. The revised manuscript has significantly improved.

Open Access This Peer Review File is licensed under a Creative Commons Attribution 4.0 International License, which permits use, sharing, adaptation, distribution and reproduction in any medium or format, as long as you give appropriate credit to the original author(s) and the source, provide a link to the Creative Commons license, and indicate if changes were made.
In cases where reviewers are anonymous, credit should be given to 'Anonymous Referee' and the source.
The images or other third party material in this Peer Review File are included in the article's Creative Commons license, unless indicated otherwise in a credit line to the material. If material is not included in the article's Creative Commons license and your intended use is not permitted by statutory regulation or exceeds the permitted use, you will need to obtain permission directly from the copyright holder.
To view a copy of this license, visit https://creativecommons.org/licenses/by/4.0/

We therefore invite you to revise and resubmit your manuscript, taking into account the points raised. In particular, please provide more detailed Methods and address the concerns of Reviewer #1 regarding the possibility of sample contamination and/or vertical transmission. We also encourage you to explore the possibility of including an SEM (or some other analysis) to better understand how infection might influence the mycobiome, or vice versa, as suggested by reviewers. Please note that while such analysis would be welcomed in revision, it would not be mandated if the reviewer concerns could be addressed in another way. Reviewer #1 also suggests some expanded discussion, particularly related to vertical transmission.

Answer: We thank the editor and both reviewers for their constructive comments and continued support. We are grateful for the opportunity to revise our manuscript and have addressed all concerns in the detailed point–by–point response below. In particular:

1. We have clarified our use of field, PCR and extraction negative controls and explained how we identified potential environmental and buffer contaminants using the decontam package.
2. We provided additional evidence that inner eggshell communities are distinct from the nest environment, including the key observation that hatched eggs are more dissimilar to the nest than unhatched eggs – contrary to what would be expected under environmental contamination.
3. We acknowledged that our cross–sectional design limits inference about directionality between infection and microbiome composition, and explained why structural equation modeling (SEM) would not overcome this limitation given our study design. We now explicitly state in the Discussion the need for longitudinal and experimental approaches to establish causality (lines 476–478).
4. We now introduce (lines 72–73) and discuss (lines 327–330) vertical transmission of the egg microbiome.
5. We have worked on tightening the writing of the manuscript throughout.

We believe these revisions address all of the reviewers' comments and concerns.

Reviewers' comments:

Reviewer #1 (Remarks to the Author):

Carranco et al. present “Turtle egg microbiome modulates fusariosis fungal infection and hatching success” for review. This manuscript presents novel hypothesis driven research

examining the relationships between the bacteriome, mycobiome, and Fusarium infection status in Podocnemis unifilis eggs over developmental time. The manuscript is generally well written. My only major concern is methodological. Namely how internal egg samples/swabs are taken; if it the same as papers cited, then it seems that the inside of the shell is swabbed after breaking/hatching, which seems to risk sampling external microbes (air, etc.) and not any or only a partial internal microbiome (please clarify if this is not correct). This method and all controls used are therefore important to address as there remains skepticism in the microbiome community of microbes existing/thriving in places like the inside of a reptile egg. Also, if there are truly microbes inside the egg and the egg shell is impermeable to microbes, where are the microbes coming from initially? If from inside the mother, then this seems vertical transmission is likely.

Answer: We thank the reviewer for their positive assessment of our manuscript. Concerning the methods used for sampling the eggs, here we address each concern below:

- All successfully hatched eggs were sampled immediately after the hatchling left the egg to avoid any contamination from the nest environment, whilst the unhatched eggs (those that failed to hatch) were opened, and the inner eggshell was swabbed immediately. This is stated in lines 509–512 in the Materials and Methods, and lines 43–48 in the SI.
- To control for environmental contamination during fieldwork, we collected a field blank by swabbing the ambient air for approximately one minute, thereby capturing potential airborne contaminants. These field blanks were included alongside PCR and extraction blanks for sample preparation and sequencing. During bioinformatic preprocessing, we used the decontam package to identify and remove taxa found in negative controls (field, PCR and extraction blanks) from all biological samples as described in lines 563–565 in the main manuscript and lines 124–128 in the SI. We have additionally clarified how field blanks were collected in lines 520–522: "To control for environmental contamination from airborne sources, field blanks were collected by swabbing ambient air for one minute." and "...decontam::isContaminant (72) function was applied using the "prevalence" method to identify and remove contaminant sequences detected in field, PCR and extraction blanks".
- Additionally, we collected samples from the nest environment (nest sand). We observed that inner eggshells and nest environment microbial communities were distinct. Crucially, hatched eggs, which are more prone to environmental contamination because the hatchling breaks the shell, were more distinct from the nest environment than carefully dissected unhatched eggs. This pattern is the opposite of what would be expected if environmental contamination were driving our results, and provides strong evidence that the microbial communities we characterised are genuine inner eggshell communities. For the mycobiome, the distinction between inner eggshells and the nest environment was weaker, likely driven by the low diversity of fungal taxa on the inner eggshells. We have now added this information to the results in lines 195–205.
- Turtle eggshells are more porous than avian eggshells, and become thinner and more permeable during development as a consequence of calcium resorption. This increased permeability aligns with our observation of increasing microbial diversity on

the inner eggshell over developmental time. We have added this information to the discussion (lines 335–337).

- Vertical transmission during oviposition is a likely route: eggs are coated with maternal cloacal microbiota as they pass through the cloaca, a mechanism demonstrated in other reptiles (Li et al. 2022; Bunker et al. 2021). We have added this to the introduction in lines 70–73 and to the discussion in lines 327–330.

We believe that, taken together, the above points provide strong evidence that the observed microbial diversity on inner eggshells is not driven by environmental contamination but instead represents a genuine and potentially functional microbiome.

There are also a few minor clarifications, suggestions, and edits that are outlined below:

Figure 1 should be changed/altered as it is partly the same figure from https://onlinelibrary.wiley.com/doi/full/10.1111/mec.16548

Answer: We have now modified Figure 1.

Line 28-29: Consider "development" or some derivative in keywords as the developmental component of this work seems important.

Answer: We have now added "embryonic development" to the keywords.

Line 69-73: Is there a turtle microbiome paper showing possible vertical transmission that could be cited here? It seems possible that this is happening in your system, although that wasn't examined. I think this possibility should be highlighted in discussion.

Answer: We now include a study on turtles in lines 70–73. "A recent study in the lizard Sceloporus virgatus demonstrated that bacteria vertically transmitted via the maternal cloaca colonise the eggshell, enhance resistance to fungal infections and promote hatching success (17). Vertical transmission has also been observed in turtles (18), but the role of the eggshell microbiome in pathogen resistance remains unknown in this taxon."

Line 79: Is it possible some of the bacteria or fungi are contaminants from air, the outside of shell, or sampling equipment? How was this controlled for (in methods)?

Answer: We thank the reviewer for raising this important point. We address contamination control in detail above. Briefly, we controlled for contamination using field, PCR and extraction blanks, and the decontam package during bioinformatic preprocessing. Additionally, inner eggshell microbial communities were distinct from the nest environment, and hatched eggs, which are more prone to contamination, were more distinct from the nest than unhatched eggs, the opposite of what would be expected if contamination were driving our results. We have clarified this in the revised manuscript (lines 195–205).

Line 98: "100-120 days" should have an en dash, make sure to use throughout

Answer: Thank you for spotting this, this has been changed throughout the manuscript.

Line 105-108: Maybe mention here or in methods that keeping nest as a random effects will help to deal with the issue of pseudoreplication (multiple eggs coming from the same nest). Based on your method of sampling, no individual egg was ever sampled more than once, correct?

Answer: Each egg was sampled only once; no individual egg was ever sampled more than once. To account for pseudoreplication arising from multiple eggs originating from the same nest, nest ID was included as a random effect in all statistical models. We have clarified this in lines 115–116 of the Results section and in lines 190–193 of the extended methods section in the SI.

Line 123-125: You might try structural equation models (SEMs) to assign directionality to the effect.

Answer: We thank the reviewer for this suggestion; however, SEMs cannot infer directionality from the data we have. Regardless of the statistical approach, our cross–sectional design compares infected and uninfected eggs at a single time point. This allows us to identify associations between infection status and microbiome composition, but cannot distinguish whether mycobiome diversity confers resistance to infection or whether infection reduces diversity. Resolving this directionality would require longitudinal sampling to compare microbiome composition before and after infection (see Gillingham et al. 2025). Unfortunately, this is beyond the scope of this paper, as it would require experimental manipulation that is difficult to achieve in this protected species. However, we have specifically addressed this limitation in the discussion lines 476–478: "Moreover, understanding the causal mechanisms underlying microbiome–pathogen interactions in eggs will require longitudinal and experimental approaches capable of disentangling the direct effects of infection on microbiome composition from host–mediated responses (67). "

Line 188-190: The nest looks different (including significantly different in beta diversity dispersion) in various panels of supp figure S1. So can you explain more how this figure supports the non-nest findings? Or clarify what you are saying here.

Answer: We thank the reviewer for raising this point. We have now clarified in more detail our interpretation of nest environment samples in lines 195–205:

"As the nest environment can influence the egg microbiome (20), we repeated all beta diversity analyses, including the nest environment microbiome, yielding quantitatively equivalent results to those presented above (SI Appendix, Fig. S2). Although the nest is an important source of the inner egg microbiome, nest microbial communities remained distinct from the inner eggshell microbiome at all development stages. These results are consistent with our previous findings in the same population (20) and are likely driven by host-mediated filtering processes. Notably, hatched eggs, which were opened by the emerging hatchling and thus more prone to environmental contamination, had a more distinct bacteriome from the nest environment than that of unhatched eggs, which were carefully dissected (SI Appendix, Fig. S2). If environmental contamination were driving the observed patterns, hatched eggs would be expected to closely resemble the nest environment, not less. This provides strong evidence that environmental contamination is not responsible for the observed patterns. In contrast,

mycobiome composition of the eggshells was less distinct from the nest environment, likely reflecting the low diversity of fungal taxa present in the inner eggshells.”

We hope this new section now clarifies our interpretations and addresses the reviewer’s comments. Inner eggshell microbiomes are indeed distinct from the nest environment microbiomes, particularly for the bacteriome, likely due to host filtering mechanisms, such as selective filtering through the eggshell barrier and different local environmental conditions within the egg, including pH and temperature. Host filtering also likely drives lower variability in microbiome composition among eggs than among nest environment samples.

Line 296: I think the discussion is good overall, but I would consider adding one or two sentences about possible vertical transmission in your system, maybe as a point to be followed-up on. Especially in Line 315-318, is it possible there is an external shell microbiome that is vertically transferred and then inherited upon hatching?

Answer: Thanks for the comment. We have now discussed vertical transmission in lines 327–330.

Line 340-342: The microbial ecology theory application to Anna Karenina is well-placed and is appreciated.

Answer: Thank you. We appreciate this comment.

Line 363-364: This would seem to align well with vertical transmission/holobiont theory.

Answer: We now discuss this possibility in more detail in lines 378–381.

Line 451: Where do you think Fusarium is originating in turtles? Was it found in your nest samples also (external environment)?

Answer: We tested for Fusarium in the nest samples, and some samples tested positive, indicating that the pathogen may be present in the nest environment. We infer that Fusarium originates from the nest environment and develops during incubation, consistent with observations in other turtle species (https://www.tandfonline.com/doi/full/10.1080/21501203.2019.1710303). We added this in the discussion in lines 454–456.

Line 534-535: Were positive PCR controls also included? For example the Zymo microbial community DNA? Or some known amount of microbial DNA?

Answer: We have now added more details concerning controls and how they were treated during bioinformatic processing in lines 563–565 in the main manuscript and in lines 124–128,159–161 in the SI. We did not include Zymo microbial community DNA or any form of positive controls.

Line 564: What were the max reads and average reads per sample? Was rarefaction used or not? I would state explicitly one way or the other so it is clear.

Answer: We thank the reviewer for this comment and apologise for not including this information in our main manuscript. We added the read count and the average per sample to the relevant section of the results (lines 94–104). In addition, we wrote the following in the relevant section of the methods to explain our decision not to rarefy our dataset (lines 566–570; 587–591):
For the bacteriome, "Preliminary rarefaction curve analysis indicated that ASV richness plateaued at sequencing depths of 10,000 reads and above (Fig. S7a). Sequencing depth was not a significant predictor of bacteriome alpha diversity (species richness: df = 1, $\chi2$ = 1.526, p–value = 0.217; Shannon diversity: df = 1, $\chi2$ = 3.530, p–value = 0.060). As all samples exceeded this threshold, we retained the full dataset without rarefying to maximize detection of rare taxa ".
For the mycobiome, "Preliminary rarefaction curve analysis indicated that ASV richness plateaued at sequencing depths of 3,500 reads and above (Fig. S7b). Sequencing depth was not a significant predictor of mycobiome alpha diversity (species richness: df = 1, $\chi2$ = 1.155, p–value = 0.283; Shannon diversity: df = 1, $\chi2$ = 0.590, p–value = 0.442). As all samples exceeded this threshold, we retained the full dataset without rarefying to maximize detection of rare taxa ".

In supplemental methods line 115-116, you use the Silva database. Consider using Greengenes2 as this is much improved over Silva version 132. https://www.nature.com/articles/s41587-023-01845-1

Answer: We thank the reviewer for this suggestion. We are aware that Greengenes2 has improved over Silva. At the time we conducted these analyses, Greengenes2 was not yet available. Importantly, our main findings are based on community–level analyses (beta diversity, differential abundance) rather than fine–scale taxonomic assignments, and are therefore unlikely to be substantially affected by the choice of reference database. Differences between Silva and Greengenes2 primarily concern lower-level taxonomic classification, whereas our key results - compositional differences between hatched and unhatched eggs, and between infected and uninfected eggs - are driven by broad community–level patterns that are robust to database choice. We will, however, take this recommendation into account in future work.

Reviewer #2 (Remarks to the Author):

The study investigates whether the inner egg microbiome (bacterial and fungal communities) of the yellow-spotted Amazon river turtle influences susceptibility to Fusarium fungal infection and hatching success by comparing infected vs uninfected eggs across developmental stages.

Good job to authors for a very careful experimental design! I understand the limitation of using only 16S and ITS markers for the functional study. With the design, a metagenomic approach would definitely helps to advances the understanding of this research (Maybe future work? :P)

Answer: We thank Reviewer #2 for their positive assessment of our experimental design and for their constructive suggestions throughout. We agree that a metagenomic approach would substantially advance our understanding of the functional mechanisms underlying microbiome–pathogen interactions in turtle eggs, and we have noted this as an important direction for future research in the Discussion (lines 396–398; 429–432).

General comments:

1. Infected samples, no matter ED or LD, were collected after FSSC-infected symptoms had developed, which means authors are comparing community diversity of healthy vs. diseased eggs at post-infection. Does it also means authors are assumming pre-infected eggs also already had lower diversity? Hence, it is unclear whether the observed microbiome differences arise early and influence outcomes, or emerge later as a consequence of embryo death, infection progression, or hatching.

Answer: We thank the reviewer for this important point, and we agree that caution is needed when inferring causality from our cross–sectional design. As noted by the reviewer, our sampling compares infected and uninfected eggs at a single post–infection time point, meaning we cannot definitively determine whether lower microbiome diversity precedes infection or arises as a consequence of it. We have explicitly acknowledged this limitation in the Results lines (134–135) and now more explicitly in the Discussion (lines 476–478), where we state: 'Moreover, understanding the causal mechanisms underlying microbiome–pathogen interactions in eggs will require longitudinal and experimental approaches capable of disentangling the direct effects of infection on microbiome composition from host–mediated responses (67).' We also note that a metagenomic approach, as the reviewer suggests, would be a valuable complement to amplicon–based methods in future work, enabling functional characterisation of the microbiome's role in pathogen resistance (lines 396–398). However, we would highlight that several lines of evidence in our study are consistent with the microbiome playing a protective role rather than simply reflecting post–infection dysbiosis, including the association between higher diversity and hatching success independently of infection status, and the identification of specific taxa with known antifungal properties in higher relative abundance in uninfected eggs.

2. On a conservation practice perspective, with your study results, can you suggest what is the next step (application or research) that may helps improving the hatchability of this turtle in the artificial incubation settings?

Answer: Thank you for this comment. We agree that this is an important point. We have expanded the Discussion (lines 485–4893) to address potential conservation applications. A promising next step would be to identify key microbial species and their antifungal compounds, with the long–term goal of developing probiotic treatments for artificial nesting sites. For example, in artificial nests, inoculating incubation substrates with host–associated microbial symbionts might help to reduce the growth of fungal pathogens. As we mentioned in our discussion, a metagenomic approach would be particularly valuable here, enabling functional characterisation of the microbial communities beyond what 16S and ITS metabarcoding can achieve, and helping to identify the specific mechanisms and metabolites involved in pathogen suppression.

Introduction:

Line 57: Last sentence. I would suggest to describe specifically about "host-protective traits" (i.e. host at the egg stage) .

Answer: We have now changed this sentence to “Moreover, the host–protective traits of turtle eggs that may confer resistance to fungal infections remain to be elucidated” in lines 56–58.

Methods:
Line 480: Just curious if the sand "controlled environment" for keeping the eggs is sterilized? If no, how do you think sand from the hatchery environment will affect your result?

Answer: The sand was not sterilised; eggs were incubated in sand from the natural nest environment, as described in lines 497–498 in the main manuscript and lines 30–32 in the SI. This was a deliberate choice to maintain natural conditions and exposure to environmental microbiomes. As we show in our previous study on the same population (Carranco et al., 2022, Molecular Ecology), the sand nest is an important source of the egg microbiome, with microorganisms from the environment colonising the inner eggshell through the permeable shell. However, although the nest is a partial source, the inner eggshell communities in this study were consistently distinct from those of the nest sand (lines 195–205), suggesting that host–filtering processes shape which microorganisms establish on the inner eggshell. Using sterilised sand would have eliminated this natural microbial source and likely altered the egg microbiome assembly process, making results less representative of natural conditions.

Line 551: What was the "significant consequences" of the ITS region length variation here means?

Answer: We clarified this sentence with the following “Because the ITS region varies substantially in length (700–900 bp), which complicates the standard DADA2 filtering and trimming workflow in QIIME2, we pre–processed the sequences in three steps.” in lines 574–576.

Line 554: cutadapt spelling error

Answer: Thank you, we have corrected the spelling error.

Line 572: "we only took the information of the infected samples with an FSSC ASV count greater than one and added it as a variable..." Can you explain again what this sentence means? I do not quite get it.

Answer: We clarified this sentence with the following lines (595–601):
For the taxonomic assignment, we built a database from NCBI GenBank using RESCRIPt (76) within QIIME2 and trained it on the specific TEF primers specific to this study, as described in (8). Only sequences assigned to Fusarium or the teleomorph names (Gibberella and Nectria) and longer than 300 bp were retained for further analysis. FSSC infection status and ASV abundance data (counts of F. solani and F. keratoplasticum) from infected samples were then added as variables to the bacteriome and mycobiome metadata for subsequent statistical analyses. Individuals were considered infected if at least one FSSC ASV sequence was detected.

For paragraph Taxonomic assignment of Fusarium (FSSC) pathogens in Line 566: Maybe it's also best to mention that ASV is look at the FSSC level instead of each species (Ff, Fk, Fs) because their sequences are too similar etc., as an additional information for readers not familiar with this taxa

Answer: See answer above. We now clearly state what we consider to be FSSC ASVs. i.e. sequences belonging to *F. solani* and *F. keratoplasticum*.

I think Line 175, sentences from the supplementary file: "Only sequences assigned to Fusarium or the teleomorph name (Gibberella and Nectria) longer than 300 were selected for further analysis" is important information to be included to the method in the main article. Consider including it?

Answer: This information is now added in the main manuscript in lines 597–598.

Supplementary file:
Almost all supporting figures are too difficult to read.

Answer: We thank the reviewer for this observation. We have increased the font for relevant figures (i.e. Figures S3 to S6).

We highly appreciate the thoughtful comments of both reviewers!
